# Cancer signature ensemble integrating cfDNA methylation, copy number, and fragmentation facilitates multi-cancer early detection

**DOI:** 10.1038/s12276-023-01119-5

**Published:** 2023-11-01

**Authors:** Su Yeon Kim, Seongmun Jeong, Wookjae Lee, Yujin Jeon, Yong-Jin Kim, Seowoo Park, Dongin Lee, Dayoung Go, Sang-Hyun Song, Sanghoo Lee, Hyun Goo Woo, Jung-Ki Yoon, Young Sik Park, Young Tae Kim, Se-Hoon Lee, Kwang Hyun Kim, Yoojoo Lim, Jin-Soo Kim, Hwang-Phill Kim, Duhee Bang, Tae-You Kim

**Affiliations:** 1 IMBdx Inc., Seoul, 08506 Republic of Korea; 2 Department of Chemistry, Yonsei University, Seoul, 03722 Republic of Korea; 3 Cancer Research Institute, Seoul National University, Seoul, 03080 Republic of Korea; 4 Seoul Clinical Laboratories Healthcare Inc., Yongin-si, Gyenggi-do 16954 Republic of Korea; 5 Department of Physiology, Ajou University School of Medicine, Suwon, 16499 Republic of Korea; 6 Division of Pulmonary and Critical Care Medicine, Department of Medicine, Stanford University, Stanford, CA USA; 7 Division of Pulmonary and Critical Care Medicine, Department of Internal Medicine, Seoul National University Hospital, Seoul, 03080 Republic of Korea; 8 Department of Thoracic and Cardiovascular Surgery, Seoul National University Hospital, Seoul, 03080 Republic of Korea; 9 Division of Hematology-Oncology, Department of Medicine, Samsung Medical Center, Sungkyunkwan University School of Medicine, Seoul, 06351 Republic of Korea; 10 Department of Health Sciences and Technology, Samsung Advanced Institute of Health Sciences and Technology, Sungkyunkwan University, Seoul, 03063 Republic of Korea; 11 Department of Urology, Ewha Womans University Seoul Hospital, Seoul, 07804 Republic of Korea; 12 Department of Internal Medicine, Seoul Metropolitan Government Seoul National University Boramae Medical Center, Seoul, 07061 Republic of Korea; 13 Department of Internal Medicine, Seoul National University Hospital, Seoul, 03080 Republic of Korea; 14 Department of Molecular Medicine and Biopharmaceutical Sciences, Graduate School of Convergence Science and Technology, Seoul National University, Seoul, 08826 Republic of Korea

**Keywords:** Cancer screening, Epigenomics, Machine learning

## Abstract

Cell-free DNA (cfDNA) sequencing has demonstrated great potential for early cancer detection. However, most large-scale studies have focused only on either targeted methylation sites or whole-genome sequencing, limiting comprehensive analysis that integrates both epigenetic and genetic signatures. In this study, we present a platform that enables simultaneous analysis of whole-genome methylation, copy number, and fragmentomic patterns of cfDNA in a single assay. Using a total of 950 plasma (361 healthy and 589 cancer) and 240 tissue samples, we demonstrate that a multifeature cancer signature ensemble (CSE) classifier integrating all features outperforms single-feature classifiers. At 95.2% specificity, the cancer detection sensitivity with methylation, copy number, and fragmentomic models was 77.2%, 61.4%, and 60.5%, respectively, but sensitivity was significantly increased to 88.9% with the CSE classifier (*p* value < 0.0001). For tissue of origin, the CSE classifier enhanced the accuracy beyond the methylation classifier, from 74.3% to 76.4%. Overall, this work proves the utility of a signature ensemble integrating epigenetic and genetic information for accurate cancer detection.

## Revolutionizing Cancer Research

A new platform, AlphaLiquid® Screening, offers simultaneous analysis of whole-genome methylation, copy number, and fragmentomic patterns in a single assay for early cancer detection. The platform utilizes a multi-feature cancer signature ensemble classifier (CSE) that outperforms single-feature classifiers. At 95.2% specificity, the CSE achieved 88.9% sensitivity for cancer detection, a significant improvement over individual classifiers. The CSE model also demonstrated improved accuracy in predicting the tissue of origin for detected cancers. This study highlights the potential of integrating epigenetic and genetic information to enhance the accuracy of cancer detection using cell-free DNA sequencing data.

This summary was initially drafted using artificial intelligence, then revised and fact-checked by the author.

## Introduction

Despite many technological advances in precision oncology, cancer is still the leading cause of mortality worldwide, especially when identified in the late stages^1^. This issue urges the development of timely and easily repeatable cancer screening approaches. Many countries have implemented nationwide cancer screening programs; for example, in South Korea, the National Cancer Screening Program was launched in 1999 to deliver free-of-charge screening for stomach, breast, and cervical cancer for Medical Aid beneficiaries^2^. However, population-wide screening is currently recommended for only a few cancers, and some existing approaches lack adequate performance or may have unnecessary risks associated with their application, such as perforation of the bowel after a colonoscopy, excessive radiation exposure, and risk of infection or bleeding^3^.

Liquid biopsies, especially cell-free DNA (cfDNA) sequencing, have gained enormous attention in the context of developing a noninvasive diagnostic tool to identify early cancer signals^4,5^. From a simple blood draw, which can be periodically obtained without much harm to the donor, various analytes could potentially be studied for real-time cancer monitoring^6–9^. Among these, circulating tumor DNA (ctDNA) embedded in cfDNA has repeatedly demonstrated its utility in identifying a minuscule amount (<0.001%) of cancer signals from early tumors^5,10,11^. In many cases, the application of deliberate machine learning combined with a large-scale feature set guided by biological insights has been essential in achieving high sensitivity and specificity^12^. Because cancer screening targets an asymptomatic population that has a low cancer prevalence (usually <5%) but a large population size, high specificity is a prerequisite for clinically applicable tests. Furthermore, for meaningful follow-up, screening necessitates the prediction of the tumor origin when cancer is suspected.

Many previous or ongoing clinical trials have involved searching for cancer signatures in cfDNA via diverse feature types: mutational signatures, copy number variations, structural variations, DNA fragmentation, or methylation changes^13^. Aberrant methylation patterns or fragmentomic profiles have been most heavily employed to develop screening tests. Because aberrant DNA methylation patterns occur early in cancer pathogenesis and each cfDNA maintains the DNA methylation states of its cell of origin^14–17^, ctDNA methylation signatures can serve as a sensitive tool for disease surveillance in patients with early-stage cancer^18–26^. The fragmentomic characteristics of ctDNA are different from those of cfDNA from normal cell origins^27–30^, and many studies have successfully applied the differences in fragment size profile, end motif patterns, or preferred end positions for early cancer detection^31–35^ or cancer subtyping^22,36,37^. In addition, (normalized) fragment coverage variation in cfDNA reflects copy number alterations in tumors, which has demonstrated utility in cancer detection^38,39^.

However, most studies have focused only on one aspect of epigenetic or genetic signatures, using targeted methylation panels assessing only predetermined sites or whole-genome sequencing data analyzing genetic variations. When possible, an integrative approach utilizing the strengths of both epigenetic and genetic information would be ideal for identifying early cancer signals on the background of extremely low concentrations of ctDNA in the bloodstream. Recent studies^38,40^ have explored multifeature approaches using whole-genome bisulfite sequencing and/or whole-genome sequencing to improve cancer detection performance.

Here, we introduce a cancer screening platform that enables simultaneous analysis of whole-genome methylation, copy number, and fragmentomic patterns in a single assay. Using a total of 950 plasma samples (361 healthy controls and 107 colon, 113 liver, 238 lung, and 131 prostate cancer patients) and 240 tissue samples, we generated whole-genome methylation sequencing (WGMS) data, which provides not only base-pair resolution of methylation status but also largely intact ctDNA fragmentomic profiles preserved during enzymatic methylation conversion^41^. We have demonstrated and highlighted cancer signatures in three feature types: genome-wide methylation patterns, copy number variations, and fragmentation profiles. Biomarker discovery was performed to identify effective methylation sites characterizing cancer-specific or tissue-specific signatures. Classifiers were then constructed to detect cancer signals and search the tissue of origin (TOO) using individual features and integrating cancer signatures from multiple features, referred to as a cancer signature ensemble (CSE). Finally, a performance improvement from using a multifeature CSE compared to using single-feature models was demonstrated.

## Materials and methods

### Study design and population

Blood, plasma, tumor tissue, and adjacent normal tissue samples of cancer patients and/or healthy controls were collected from multiple sources. All procedures were approved by the institutional review boards at the local institutions (Supplementary Table 1). The inclusion criteria for cancer participants were as follows: older than 18 years and diagnosed with colorectal cancer, hepatocellular carcinoma, any type of lung cancer, or prostate cancer. Patients with two or more primary cancers at the same time or comorbidities were not included in the study (when the related information was available). Blood or plasma samples from cancer patients and associated demographic information were based on the condition before patients received any treatment. Cancer stages were defined using the American Joint Committee on Cancer Staging Manual, 8th edition^42^. For healthy controls, all participants were required to be at least 18 years of age, and individuals who were diagnosed with cancer after sample collection or who were treated with cancer therapy or showed any symptoms of cancer or were pregnant or received organ transplantation were not included in the study.

### Sample preparation and whole-genome methylation sequencing (WGMS)

Each blood sample received was centrifuged in Ficoll solution at 1500 × *g* for 15 min, and the plasma was transferred from the separated blood. Plasma was separated by centrifugation at 16,000 × *g* for 10 min to remove cell debris. Plasma and fresh frozen tumor tissues that were sent to IMBdx directly followed subsequent steps starting from DNA extraction. cfDNA was extracted from 1–10 mL of plasma using the Maxwell® RSC ccfDNA Plasma Kit (Promega, USA) according to the manufacturer’s instructions. After extraction, cfDNA was quantified using the Cell-free DNA ScreenTape Assay and the 4200 TapeStation System (Agilent, USA). Tumor tissue genomic DNA was extracted from frozen fresh samples using the Maxwell® RSC Tissue DNA Kit (Promega) according to the manufacturer’s instructions. Extracted tumor tissue genomic DNA was quantified using a Qubit dsDNA High Sensitivity Kit (Thermo Fisher Scientific, USA) and fragmented using an ultrasonicator instrument (Covaris, USA) according to the manufacturer’s instructions.

Methylation sequencing library preparation was carried out as per the IMBdx AlphaLiquid^®^ Screening platform protocol. The prepared libraries were sequenced using the NovaSeq 6000 platform at 2 × 150 bp to generate ~100 Gb of raw sequencing data.

### NGS preprocessing and quality control

FASTQ files were generated from binary base call (BCL) sequence files using Illumina bcl2fastq software (version 2.20.0.422). Illumina-specific adapters and low-quality sequences (-q 20 -u 20 -x -y -3 -p -g -t 1 -T 1) were trimmed using fastp (version 0.23.1)^43^. The sequenced reads were aligned to the human genome (GRCh37) using bitmapperBS (version 1.0.2.1)^44^. The aligned BAM files were sorted and indexed using SAMtools (version 1.11)^45^. PCR and optical duplicates were marked and removed using GATK tools (version 4.2.3.0) MarkDuplicates^46^, generating a final BAM file for feature extraction.

To check the quality of the results, we calculated the uniquely mapped reads, mapping rate, and duplication rate using SAMtools. Conversion efficiency was calculated using MethylDackel methylation reports (https://github.com/dpryan79/MethylDackel).

The exclusion criteria for quality control of WGMS data included uniquely mapped reads of less than 150 million, a mapping rate of less than 80%, a duplication rate of more than 25%, and a C-to-T conversion efficiency of less than 99%.

### Definition of methylation regions in WGMS (CpG blocks)

The human reference genome (GRCh37) from Ensembl was scanned to obtain all CG dinucleotide sites, resulting in 28,245,162 CpG sites. Segments containing three or more contiguous CpGs with a neighboring distance within 100 bp and having analyzable coverage (median coverage at each CpG site in the healthy training cohort, >3×) were identified. Segments longer than 1000 bp were divided into two or more similar-sized regions that were smaller than 1 kb. Those belonging to X and Y chromosomes or included in the ENCODE blacklist^47^ were not included. The remaining 2,488,047 regions constituted our final set and were used for marker selection and classification. The final set contained 19,289,514 CpG sites^48^ and spanned 522 Mb.

### Methylation level quantification of WGMS data

For WGMS data, the methylation level in each methylation region was quantified as the AMF, which was calculated using all nonduplicated reads mapped to that region. Specifically, it was calculated as:

$${AM}{F}_{i}=\frac{{\sum }_{j\in {R}_{i}}{C}_{j}}{{\sum }_{j\in {R}_{i}}({C}_{j}+{T}_{j})},$$

where ${R}_{i}$ denotes the set of all CpG sites in region $i,$ and ${C}_{j}$ and ${T}_{j}$ denote the number of cytosines and thymines, respectively, covered at the $j$-th CpG in the region. In the calculation for each sample at each region, we regarded the AMF value as missing (NA, not available) if the average read coverage in the corresponding segment was less than 10×.

### Retrieval of methylation information from TCGA

TCGA Illumina Infinium HumanMethylation450 (450 K) BeadChip data were retrieved from the GDC legacy archive^49^. For each of our cancer cohorts, the related TCGA cancer cohorts were obtained: COAD (colon adenocarcinoma, *n* = 296 vs. 38 for cancer and normal tissues), READ (rectal adenocarcinoma, *n* = 98 vs. 7) for colon cancer, LIHC (liver hepatocellular carcinoma, *n* = 377 vs. 50) for liver cancer, LUAD (lung adenocarcinoma, *n* = 458 vs. 29) and LUSC (lung squamous cell carcinoma, *n* = 370 vs. 40) for lung cancer, and PRAD (prostate adenocarcinoma, *n* = 498 vs. 50) for prostate cancer.

For each methylation region analyzed in our data, the corresponding methylation level in TCGA data was obtained by searching all of the matching CpG positions in the 450 K chip and then computing the median beta values reported in the chip. Approximately 185,236 (7.4%) of our methylation regions had corresponding CpG sites (85.82%) in the chip.

### Methylation marker selection for classification

Tumor tissue, adjacent normal tissue, and healthy control cfDNA samples from WGMS data were used to select markers capturing cancer signals. For each cancer type, we aimed to identify tissue-specific markers by comparing AMF values between tissues (tumor/normal) and cfDNA samples and by comparing cancer-specific markers in tumor tissue with those in normal tissues. Marker selection was focused on regions where healthy samples were either stably unmethylated or stably methylated; specifically, regions where 90% of healthy training samples had an AMF < 0.3 were referred to as healthy-unmethylated, and those where 90% of healthy training samples had an AMF > 0.7 were referred to as healthy-methylated.

Differential methylation analysis was performed using the R “limma” package^50^. For healthy-unmethylated regions, we compiled the top 10,000 significantly differentially methylated markers (log FC > 1.0 and FDR < 0.01) that had (i) a higher AMF in tumor tissue than in healthy cfDNA (“T-H”), (ii) a higher AMF in normal tissue than in healthy cfDNA (“N-H”), or (iii) a higher AMF in cancer tissue than in normal tissue (“T-N”) based on WGMS data. The differentially methylated markers in TCGA tumor and normal tissues were also collected and merged into the “T-N” set. Significantly differentially methylated markers for the healthy-methylated regions were similarly found. Tissue-specific markers included those that overlapped between the “T-H” and “N-H” sets but did not overlap the “T-N” set. Cancer-specific markers included those that overlapped between the “T-H” and “T-N” sets but did not overlap the “N-H” set.

Tissue-specific and cancer-specific markers had a high proportion of overlap among healthy-unmethylated samples (21.3% intersected by all cancer types), but the overlap was minimal for healthy-methylated samples (0.97% intersected by all cancer types). Our final cancer vs. noncancer classification marker set was determined to include all healthy-unmethylated markers (*n* = 67,639) after visual inspection of the heatmap, functional category decomposition, and tradeoff assessment between classification power and stringency constraint (fewer markers with a more stringent constraint). The marker set used to trace the TOO was determined by additionally including tissue-specific markers from healthy-methylated regions (*n* = 43,338).

### Functional annotation of methylation markers

Functional annotation regarding transcription control or gene type was performed using HOMER (version 4.1)^51^. CpG islands were annotated based on the cpgIslandExt file downloaded from the UCSC table browser^48^. Gene set enrichment analysis was carried out using “enrichr”^52^. For colon, liver, lung, and prostate cancer, 5465, 2841, 4190, and 9950 cancer hypomethylated regions were retrieved, respectively, and the associated genes were searched, except for those in the intergenic regions. Cell type-related categories enriched in the Human Gene Atlas database were then analyzed.

### Characterization of copy number patterns

To reflect the existence of copy number alterations, CNRs were calculated using the R package “QDNAseq”^53^ over nonoverlapping bins at a specified bin size. Sequenced reads in each bin were counted, and bins that overlapped with the QDNAseq default blacklist (wgEncodeDacMapabilityConsensusExcludable.bed) were removed. GC bias and mappability were corrected, followed by normalization and smoothing, to generate the CNRs. Bin segmentation, normalization, and calling of copy number variation segments were carried out using default parameters.

To extract regions that frequently experienced copy number gain events, we performed differential CNR analysis on tissue samples, comparing cancer tissue and normal tissues for each cancer type. However, normal tissues did not exist for prostate cancer; thus, a composite of all normal tissues from other cancer types was used. Thresholds for retrieving frequent gain events were set as log FC > 0.2 and FDR < 0.01 for colon and liver cancers and log FC > 0.15 and FDR < 0.001 for lung and prostate cancers, resulting in 2168 markers for colon, 2680 for liver, 1603 for lung, and 1041 for prostate cancer. The list of robust copy number gains was also retrieved from the TCGA pancancer study^54^, and any of our 100-kb bins overlapping TCGA markers were extracted.

To determine the best resolution for the classification exercise, genomic binning was evaluated over a broad range, 1 kb, 5 kb, 50 kb, 100 kb, 500 kb, and 1 Mb. We examined the tradeoff between reducing noise levels and rescuing biological signals and decided to use 100 kb bins. Manual inspections of several tumor and cfDNA pairs suggested that focal tissue events were frequently lost at lower resolution, and 100 kb corresponded to the highest resolution that did not decrease the classification performance of CNRs. For classification input, an adjusted CNR was calculated using only short fragments (<150 bp) to enrich tumor-associated fragments and by treating values between 0.85 and 1.05 as copy neutral.

### Fragmentomic feature engineering

The fragment size of each read was calculated using *bamPEFragmentSize* from Deeptools (version 3.5.1)^55^. ENCODE blacklist regions were not used for fragment size analysis. To quantify fragmentomic signals, the S/L ratio was calculated for a specified bin size^31^. Short fragments were defined as having lengths between 80 and 150 bp, and long fragments were defined as having lengths between 151 and 220 bp. The short and long fragments were counted over nonoverlapping bins using *bamCoverage* from Deeptools (version 3.5.1) with the parameter option not to multiply by counting duplicated reads. The ratio was calculated by dividing the number of short fragments by the number of long fragments. The normalized S/L ratio per bin was obtained by dividing the raw ratio by the S/L ratio computed at the chromosome level. The log2-transformed value was used for visualization and classification.

The bin size was evaluated with sizes of 10 kb, 50 kb, 100 kb, 500 kb, 1 Mb, and 5 Mb. After examining the raw count of short and long fragments and coherence among normalized count profiles, we decided to use a size of 100 kb for downstream analysis.

### Model building workflow: cross-validation training, testing, machine learning, and prediction

The healthy and cancer cfDNA samples were divided into training and test sets, balancing age, sex, and cancer stage, to build a classification model and for evaluation. Healthy cohorts were divided into training and test sets within each group of people aged <50 or ≥50 years. Of note, classification model fitting and performance evaluation were only based on the group aged ≥50 years. The training portion was further split 4-fold for cross-validation performance assessment.

For a given feature type and with a given portion of the training set (75% of the entire training set for cross-validation and 100% for independent test validation), model building and score prediction were performed as follows:

1. Imputation of missing values: For methylation, missing values were imputed by the average value of the healthy controls in the training portion with no missing data. Markers that had a missing value in more than 10% of the healthy controls were discarded.
2. Machine learning: The classification model was built with multiclass labels using healthy controls and colon, liver, lung, and prostate cancer patients using a support vector machine algorithm^56^ on a vector of input features. The hyperparameter was tuned to achieve the lowest probability of misclassifying unseen test examples. The support vector classifier (SVC) function in the scikit-learn Python library was trained using a linear kernel.
3. Prediction: The prediction score was obtained for each sample in the test portion. The cancer score was obtained from a vector of five probabilities supporting the five class labels by summing the portion supporting cancer. For the TOO, the conditional probabilities supporting the four cancer types were obtained from the original probability by dividing by the cancer probability.

### Construction of multifeature ensemble classifiers

For cancer detection, an ensemble classifier was constructed by averaging cancer scores generated from three individual classifiers built on (i) AMF values of 67,639 healthy-unmethylated markers, (ii) CNRs estimated for 100-kb bins, and (iii) fragmentomic S/L ratios computed for 100-kb bins. For each individual and ensemble classifier, the score threshold was determined post hoc using the prediction scores of the test set. The lowest threshold satisfying >95% specificity was chosen. Our final classification was based on the ensemble classifier; thus, samples detected as suspected cancer by the ensemble classifier further underwent TOO classification.

For TOO classification, an ensemble classifier was constructed by averaging TOO conditional probabilities from three individual TOO classifiers built on (i) AMF values of 110,977 selected markers, (ii) CNRs estimated for 100-kb bins, and (iii) fragmentomic S/L ratios computed for 100-kb bins. For each individual and ensemble classifier, TOO prediction by default was performed based on the class corresponding to the maximum TOO probability.

### Data visualization and statistical analysis

Genome browser snapshots were generated using the Integrative Genomics Viewer^57^. UMAP analysis was performed using the Python UMAP library^58^, and PCA was performed using R (version 4.1.1) software. Student’s *t* test was used to compare continuous variables between independent groups, and McNemar’s test was used to compare the sensitivity between different classifiers. The statistical analysis was performed using R (version 4.1.1) software, and graphical visualization was performed using the “ggplot2” package. Differential methylation and copy number analyses were performed using the R “limma” package^50^. The sensitivity, specificity, AUC, and associated CIs were calculated using the R “pROC” package. Classification models were built using the SVC function in the scikit-learn Python library^59^.

## Results

### AlphaLiquid^®^ Screening assay and cohort overview

**Fig. 1:**
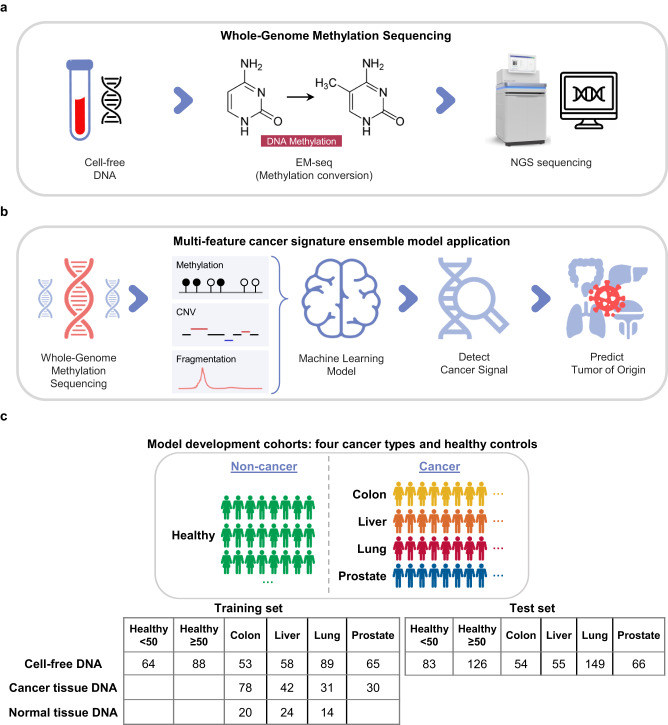
AlphaLiquid^®^ Screening workflow and cohort summary. **a** Generation of whole-genome methylation sequencing (WGMS) data. A cell-free DNA (cfDNA) sample was treated with enzymatic methylation conversion and then sequenced on a next-generation sequencing (NGS) machine. **b** Application of our multifeature cancer signature ensemble (CSE) models. After preprocessing the WGMS data, methylation, copy number, and fragment size features were extracted and fed to CSE machine-learning models for cancer signal detection and tissue-of-origin prediction. **c** Cohorts for model development and independent test performance evaluation. The samples were collected from healthy controls (noncancer, green) and patients from four cancer types: colon (dark yellow), liver (orange), lung (deep pink), and prostate (marine blue). Model training and evaluation were performed using cfDNA samples, but cancer tissue DNA or (adjacent) normal tissue DNA from cancer patients assisted feature selection. The healthy cohort was divided into two groups depending on age: older than 50 years or younger. The cfDNA samples were split into training and test sets, given a constraint that any cfDNAs with matched tissue samples were assigned to the training portion.

We developed the AlphaLiquid^®^ Screening platform for the early detection of multiple cancers. It combines enzymatic methylation conversion^41^ with whole-genome sequencing (Fig. 1a). Because unmethylated cytosines at CpG sites are converted to uracils, the base-level methylation status can be revealed genome-wide. Additionally, copy number patterns and fragmentation profiles can be explored because enzymatic conversion is expected to cause minimal fragmentation damage and coverage bias^41^. Using these features, the platform applies machine learning models to identify cancer signals and predict the TOO for those detected as cancer (Fig. 1b). Because multiple features were incorporated to characterize the underlying cancer signals, we refer to the combined signature as the CSE.

To construct CSE models and show the clinical performance of AlphaLiquid^®^ Screening, we collected plasma samples of four cancer types (colon, liver, lung, and prostate cancers) and healthy controls from multiple sources (“Materials and methods,” Fig. 1c and Supplementary Table 1). cfDNA was successfully extracted from a total of 991 (626 cancer and 365 healthy) plasma samples, which were then subjected to WGMS (Supplementary Fig. 1). On average, 100.9 Gb of raw sequencing reads were generated, covering the whole genome at approximately 30×. The samples then underwent preprocessing and quality checking (Supplementary Fig. 2). Three samples were discarded because of inadequate blood sampling, and an additional 38 (4%) were excluded due to low input DNA, a low conversion rate, a low mapping rate, or a high duplication rate (“Materials and methods,” Supplementary Fig. 1). The sequencing statistics after quality control are summarized in Supplementary Table 2. Because cancer occurs more often in the aged population, we restricted healthy controls to those older than 50 years for model construction and evaluation. However, all of the healthy samples in the training set were used for feature engineering and marker selection. For marker discovery, we additionally generated WGMS data from 181 tumors (cancer tissues) and 58 adjacent normal tissues (Fig. 1c). A portion of the tissue samples had matched plasma samples (92 and 53 samples for cancer and normal tissues, respectively). For model training and evaluation, 950 analyzable cfDNA samples were divided into independent training and test sets, balancing age, sex, and cancer stage as much as possible, given that all plasma samples with matched tissues were assigned to the training portion. The clinical characteristics of the two sets are summarized in Table 1. The clinical stage was evenly distributed in colorectal cancer but varied in other cancer types; samples were more enriched for earlier stages of hepatocellular carcinoma, enriched for stage 1 or stage 4 of lung cancer, and enriched for stage 2 and stage 3 of prostate cancer.

**Table 1:** Clinical characteristics of participants.

	Training set						Test set					
	Healthy <50 (*N* = 64)	Healthy ≥50 (*N* = 88)	CRC (*N* = 53)	HCC (*N* = 58)	LC (*N* = 89)	PC (*N* = 65)	Healthy <50 (*N* = 83)	Healthy ≥50 (*N* = 126)	CRC (*N* = 54)	HCC (*N* = 55)	LC (*N* = 149)	PC (*N* = 66)
Age, years												
Average	40.56	58.8	62.45	60.97	64.62	69.85	41.96	58.64	60.8	60.93	66.08	69.21
Range	22–49	50–76	41–88	39–86	31–82	53–84	25–49	50–78	33–79	34–86	36–83	44–84
Sex, *n*												
Male	27	29	33	47	67	65	34	34	32	42	104	66
Stage, *n*												
Stage 1			11	19	17	1			12	20	62	0
Stage 2			12	27	7	42			11	22	17	42
Stage 3			12	5	2	16			12	8	7	17
Stage 4			18	7	63	6			19	5	63	7

Within each training and test set, the clinical information is summarized for six groups: healthy controls younger than 50 years (“Healthy <50”), healthy controls older than 50 years (“Healthy ≥50”), colorectal cancer (CRC), hepatocellular carcinoma (HCC), any type of lung cancer (LC), and any type of prostate cancer (PC). “*N*” refers to the sample size.

### Cancer signature in methylation patterns

**Fig. 2:**
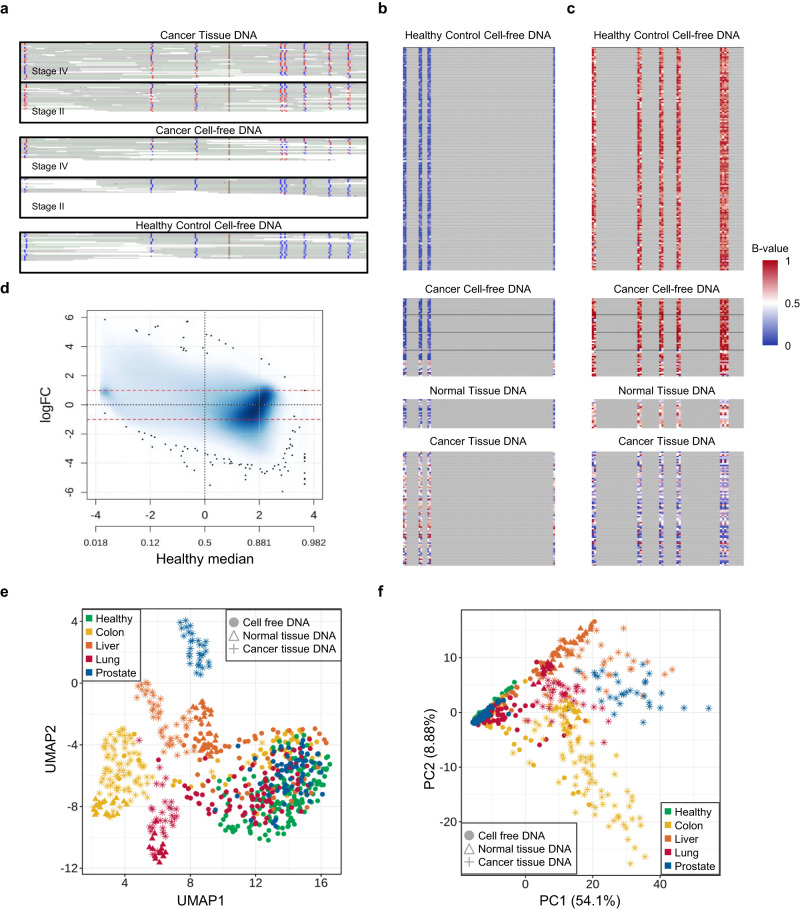
Cancer signature captured in methylation patterns. **a** IGV snapshot demonstrating base-pair level methylation information in NGS data. Five panels display the read alignment of two cancer tissue DNAs (upper two panels) and their matched cell-free DNA (cfDNA) samples (middle two) from stage 4 and stage 2 cancer patients and one healthy cfDNA sample (bottom). Red indicates a methylated read base, and blue indicates an unmethylated base. **b**, **c** Examples of co-methylated CpG sites that were hypermethylated (**b**) and hypomethylated (**c**) in colorectal cancer cfDNA relative to healthy cfDNA. For each panel (sample type), the methylated read fraction (*B*-value) is shown for each CpG site (non-gray colored columns) in each sample (rows). Non-CpG sites are in gray. **d** Smoothed scatter plot showing the overall difference in the region-level methylation value (quantified as logit-transformed average methylation fraction) between the colon cancer tissue DNA and healthy cfDNA samples. The median difference in the methylation levels (*y*-axis) is shown against the median of those in the healthy cohort (*x*-axis). All ~2.4 million methylation regions were plotted, and outliers are indicated as black dots. **e** Uniform manifold approximation and projection (UMAP) analysis of all training cfDNA and tissue samples based on ~2.4 million methylation regions. Different cohorts are indicated by color (green, healthy; yellow, colon; orange, liver; red, lung; and blue, prostate cancer), and different sample types by point type (filled circle, cfDNA; filled triangle, adjacent normal tissue; and star, tumor tissue). The first two UMAP components were plotted. **f** Principal component analysis based on the 67,639 methylation regions with low methylation in the healthy cohort.

Using WGMS data, the methylation level could be quantified at the CpG level (1 base pair (bp) resolution, Fig. 2a) or a regional level that contained several consecutive CpGs (Fig. 2b, c). Figure 2a shows an Integrative Genomics Viewer snapshot capturing one genomic region containing eight CpGs. At a single CpG site, the methylation level could be quantified as follows: at the third CpG site, the fraction of methylated reads among all aligned reads was 0% (0/23) for the healthy control group, while the values were 55% (20/36) and 56% (14/25) for the first and second tissue samples from the stage 4 and stage 2 cancer patients, respectively. Additionally, the fractions of the corresponding cfDNAs were 33% (6/18) and 0% (0/15), respectively, reflecting varying degrees of ctDNA shedding.

Because quantification of methylation at a single CpG site often results in high variability and sequencing errors, making cfDNA analysis challenging, we carried out most methylation analyses at the regional level to achieve more stable quantification. The methylation regions used are shown in the Materials and Methods and Supplementary Fig. 3. Briefly, starting with ~28 million CpG sites found in the human reference genome, three or more consecutive sites within a neighboring distance of 100 bp were considered as a segment after low-depth sites or blacklist regions were discarded. In total, ~2.5 million regions were available for downstream analysis. In our study, the methylation level was quantified as the average methylation fraction (AMF, defined as the fraction of cytosines in the included CpG positions) unless otherwise stated. Examples of differentially methylated regions (DMRs) between colon cancer cfDNAs and healthy controls are shown in Fig. 2b (cancer hypermethylated) and Fig. 2c (cancer hypomethylated).

Because our platform covers the entire genome, DMR searches can be flexible, contrasting any two groups of interest. Figure 2d displays the methylation difference between healthy and colon cancer tissue cohorts across all ~2.5 million regions. Tens of thousands of DMR candidates may exist. The figure also presents the relative abundance of cancer hypo- and hypermethylated regions. Supplementary Fig. 4a shows that methylation patterns differ between cfDNA and tissues and between tumor and normal tissues. Differences in methylation distribution arise from their distinct cell types^16^. As tumors develop and grow, their cellular diversity leads to more varied methylation patterns in tumor tissues compared to normal tissue, and the corresponding anomalies appear in cfDNA due to the shedding of cancer DNA into the bloodstream. Although small, a consistent peak at zero AMF appears for all cohorts, suggesting the existence of persistently unmethylated regions that may govern the expression of essential regulatory genes. A subset of cancer tissues and a few cancer cfDNAs exhibit global hypomethylation compared with normal tissues or healthy cfDNAs, aligning with observations in the literature^60^. The divergence level in the methylation profile (measured by the region-specific standard deviation (SD)) is often several-fold higher in a cancer tissue cohort than in a cfDNA cohort (Supplementary Fig. 4b). In healthy cfDNA and normal tissues, methylation levels are stable. In contrast, cancer tissues show notable variations due to tumor heterogeneity. These variations might be reflected in the cfDNA of cancer patients but are less pronounced due to the limited presence of ctDNA.

Unsupervised clustering by uniform manifold approximation and projection (UMAP) analysis (Fig. 2e) revealed that tissue (tumor/normal) samples were well clustered according to each TOO and then by cancer status (i.e., normal tissues were further clustered). Most cfDNAs, however, were separate from tissue clusters and formed relatively loose (with respect to cancer origin) and mixed clusters. Principal component analysis (PCA) showed a similar result but on a different scale (Fig. 2f). These unsupervised clustering results suggested that samples are reasonably clustered by cancer type and sample type. These delineations not only facilitated differentiation between cancer patients and healthy controls but also enabled accurate inference of the tissue of origin. Overall, a cancer signature was demonstrated in cfDNA samples that reflected the methylation signature in tissue samples, which differed by cancer type and cancer status.

### Methylation marker discovery

**Fig. 3:**
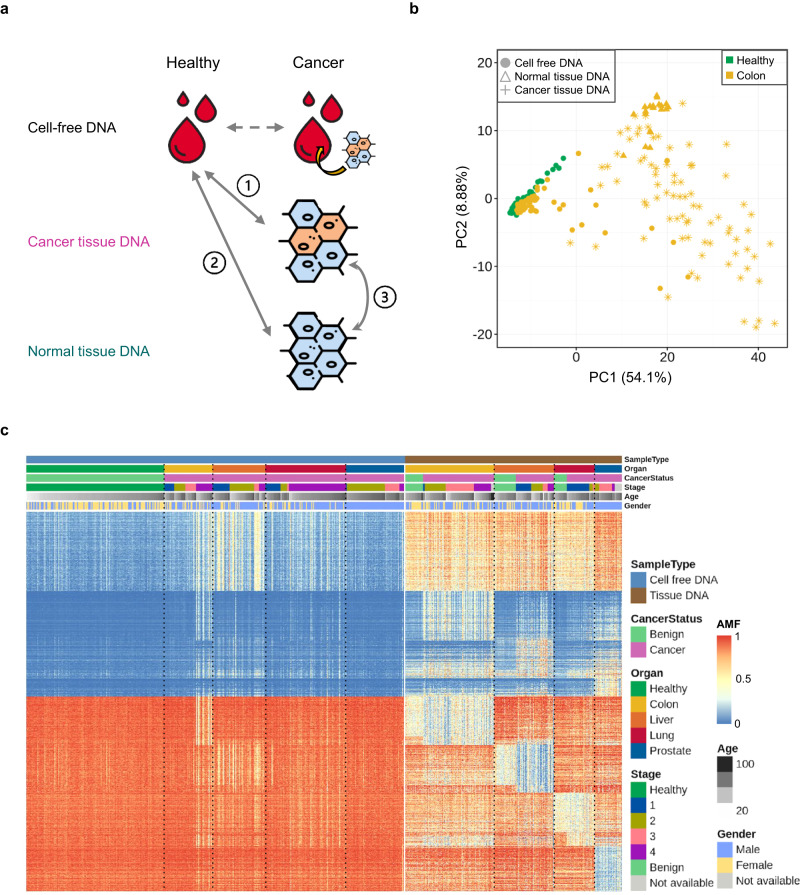
Methylation marker discovery. **a** Differential methylation analysis strategy comparing methylation levels between (i) cancer tissue DNA and healthy cell-free DNA (cfDNA); (ii) normal tissue DNA and healthy cfDNA; and (iii) cancer tissue and normal tissue DNA. **b** Principal component analysis of healthy cfDNA samples (green-filled circle) and all sample types from colon cancer (yellow-filled circle for cfDNA, yellow-star for tumor tissue, and yellow-filled triangle for adjacent normal tissue). Average methylation fractions of ~67,000 regions with low methylation levels in the healthy training set were used. **c** Heatmap displaying high-ranked differentially methylated markers that exhibit cancer-specific or tissue-specific signatures (see Materials and Methods). The annotation on the upper part of the panel indicates sample type (cfDNA or tissue), organ site (healthy, colon, liver, lung, and prostate), cancer status (benign or cancer), cancer stage (if available), age, and sex. The cells in the heatmap are colored by the associated average methylation fraction (AMF).

Taking advantage of the availability of many tissue samples, we carried out methylation marker discovery for classifiers while detecting cancer signals and localizing the TOO in parallel. First, we parsed out the variations across sample types and cancer types by comparing the methylation levels between (i) cancer tissue and healthy cfDNA (“T-H”), (ii) normal tissue and healthy cfDNA (“N-H”), and (iii) cancer tissue and normal tissue (“T-N”) (Fig. 3a) and repeated the analysis for all cancer types. Normal tissues were not available for prostate cancer; thus, a portion of the analysis was omitted. PCA of healthy cfDNAs and all samples from one cancer type at a time demonstrated differences by sample type (Fig. 3b and Supplementary Fig. 5). Cancer tissues were widely dispersed, normal tissues were clustered on the edge of the tumor space, and healthy and cancer cfDNAs were closer to each other but slightly separated.

The search for meaningful markers was focused on regions with either consistently low or high methylation in healthy controls (“Materials and methods”). We refer to the former as “healthy-unmethylated” and the latter as “healthy-methylated” regions; they served as supersets for cancer hyper and hypomethylated markers, respectively. Differential methylation analyses were conducted using WGMS and The Cancer Genome Atlas (TCGA) 450 K data (“Materials and methods,” Supplementary Figs. 6 and 7). More differentially methylated markers were found between healthy cfDNA and (tumor/normal) tissue than between cancer and normal tissue; among healthy-unmethylated regions, 28,057, 25,924, and 2670 markers were found by T-H, N-H, and T-N comparisons for colon cancer with a log fold change (FC) > 1 and false discovery rate adjusted *p* value (FDR) < 0.01. The cancer-specific signal (contrasting cancer tissue and normal tissue) was strongest for liver cancer and minimal for lung cancer in our data (Supplementary Fig. 8). The proportion of markers with an associated (absolute) log FC greater than 1.0 was 19.8%, 49.6%, and 1.8% for colon, liver, and lung cancer, respectively. A search for differentially methylated markers using cfDNA samples only (Supplementary Fig. 9) did not result in many strong candidates; 4214, 389, and 282 markers were found with a log FC > 1 and FDR < 0.01 for healthy vs. colon, lung, and prostate cancer comparisons, respectively.

After examining tissue-specific and cancer-specific markers derived from all cancer types in WGMS and TCGA data, we obtained two methylation marker sets, one for identifying cancer (entire “healthy-unmethylated” set; *n* = 67,639) and the other for determining the TOO (“TOO set”; *n* = 110,977). Figure 3c displays a subset of high-ranked markers that consist of tissue-specific or cancer-specific markers in healthy-unmethylated regions (2000 and 2676 markers from Supplementary Fig. 10a, b, respectively) and organ-specific or tumor-specific markers among healthy-methylated regions (3000 and 1927 markers from Supplementary Fig. 10c, d, respectively). Compared to all genomic regions, the TOO and healthy-unmethylated sets had a higher proportion of promoter regions (5.6%, 23.6%, and 34.9% were observed for all, TOO, and unmethylated sets, respectively) and overlapping CpG islands (1.9%, 27.0%, and 44.0% were observed for all, TOO, and unmethylated sets, respectively) (Supplementary Fig. 11a, b). Using gene set enrichment analysis of the hypomethylated regions for each cancer type, we identified the target organ-related cell type as the top hit (Supplementary Fig. 11c).

Overall, through extensive joint analysis of cfDNA and tissue cohorts, we obtained effective marker sets for cancer detection and tracing of the origin.

### Cancer signature reflected in copy number patterns

Copy number alterations frequently occur in many cancers. In cfDNA studies, alteration patterns are usually studied using whole-genome sequencing data by analyzing normalized read coverage variations^61^. Here, we investigated the cancer signature reflected in cfDNA using WGMS, which also generated next-generation sequencing (NGS) data but from a library that was subjected to enzymatic methylation conversion.

At the individual sample level, copy number ratios (CNRs) were calculated over nonoverlapping 100-kb bins (“Materials and methods”) and served as a basis to quantify the magnitude of abnormalities when distanced from zero on the log scale. The CNR profiles of several examples are shown in Supplementary Fig. 12. For colon, liver, and lung cancers, cfDNA, cancer tissue, and normal tissue were obtained from the same patients with stage 4, stage 2, and stage 1 cancer, respectively. For low-stage cancer, the variability in cfDNA was largely comparable to the noise level observed in healthy samples, at least in the visible spectrum.

**Fig. 4:**
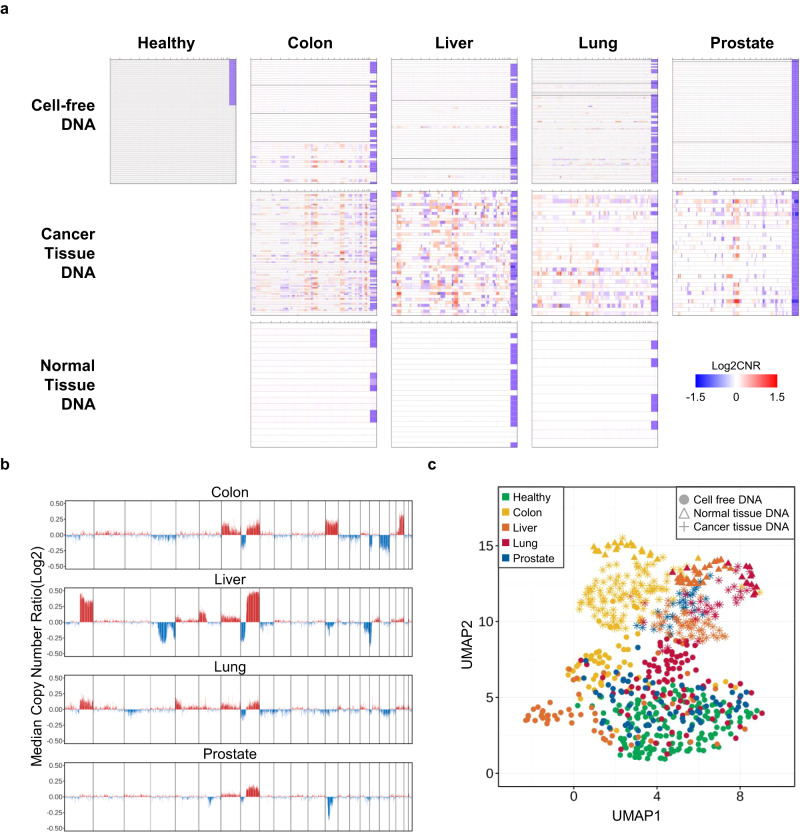
Cancer signature revealed in copy number patterns. **a** Genome-wide (log2-scale) copy number ratio (CNR) profiles of cfDNA (top row), cancer tissue DNA (middle row), and normal tissue DNA (bottom row) samples across five cohorts (columns). Within each panel, the CNR profile of each sample is shown horizontally along the genomic coordinates, displaying chromosome X at the end. The cancer samples in the cfDNA panels are ordered by cancer stage. **b** Magnitude of overall copy number alterations in the cancer tissue cohorts. The median of log2-transformed CNR was computed for each 100 kb bin using the cancer tissue samples in each cancer type (panels). **c** Uniform manifold approximation and projection (UMAP) analysis of all training cfDNA and tissue samples based on CNR values. The other graphical details are similar to those in Fig. 2e.

The cohort-level CNR profile by sample type and cancer type is shown in Fig. 4a. For all cancer types, cancer tissue cohorts demonstrated substantial copy number events (83%, 100%, 87%, and 87% of colon, liver, lung, and prostate tumors had at least one event). When the magnitude of overall copy number alterations in each cohort was summarized as the median CNR, both shared and diverged copy number patterns were observed across the different cancer types (Fig. 4b). All cancer types showed frequent gain and loss events on chromosomes 7 and 8^62^, while certain events were present only for a subset of cancer types, such as chromosome 13, where gain events frequently occurred in colon cancer but not in other cancers. In contrast to cancer tissues, adjacent normal tissues did not suggest that any copy number events had occurred (Fig. 4a). Among cfDNAs, no significant copy number events were found in healthy controls, but observations varied for cancers (upper panels in Fig. 4a). For the colorectal cancer cohort, which had a well-balanced cancer stage distribution, copy number alterations were visible for some stage 4 samples. For liver and prostate cancers, relatively few substantially large copy number alterations were observed in our cohorts.

UMAP analysis based on CNRs (Fig. 4c) revealed a broad separation between tissue and cfDNA samples, which may partially reflect the impact of different library preparation methods on fine-scale normalized read depths. However, UMAP analysis also presented clusters of tissue samples by origin and by sample type, and moderate clusters were observed within the cfDNA portion by cancer type, except for in the prostate and healthy cohorts.

Our analysis confirmed frequent copy number events in cancer tissues, but these events were visible in cfDNA of high-stage cancer patients only. Nonetheless, unsupervised clustering using genome-wide CNR values suggested the presence of fine-scale variations that could be informative in separating cancer samples from healthy control samples.

For classification purposes, we performed several modifications to improve the signal-to-noise ratio. We re-estimated CNRs using reads shorter than 150 bp to enrich ctDNA, and we suppressed the fine-scale variations that were also observed in the healthy cohort and then focused the algorithm training on the regions showing frequent copy number gains in our WGMS tissue cohorts (Supplementary Fig. 13) or in a TCGA pancancer study^63^ (Supplementary Fig. 14) (for more details, see “Materials and methods”).

### Cancer signature captured in the fragmentation profile

DNA fragments derived from tumors are known to have a different length distribution than typical fragments from the usual cell death process^28,29^. WGMS retains the maximum number of fragments that are intact from fragmentation damage^41^; thus, we were able to employ the cancer signature captured in fragmentomic patterns.

**Fig. 5:**
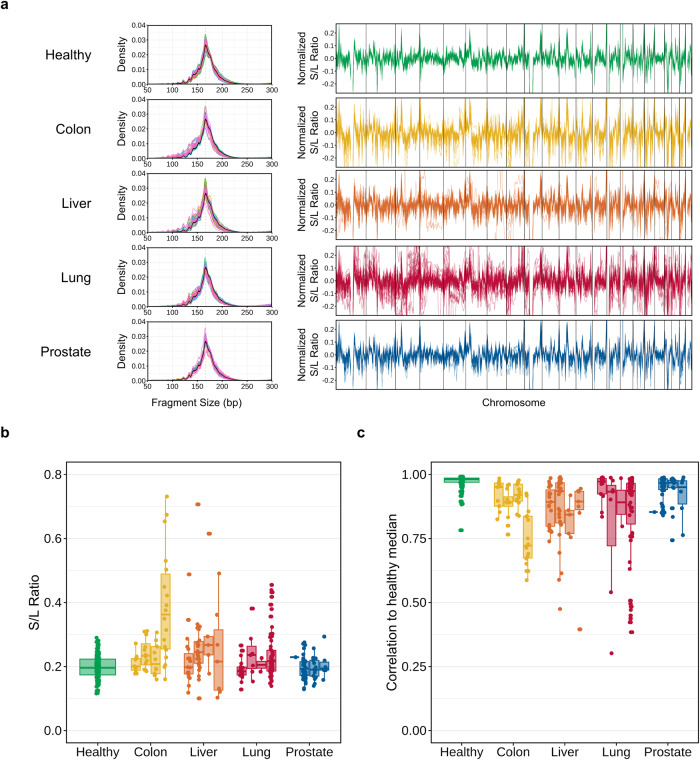
Cancer signature present in fragmentomic patterns. **a** Density plot of fragment size (left) and line plot of regional fragment size ratio (FSR) profile (right) for each of five cfDNA cohorts: healthy control and colon, liver, lung, and prostate cancer. Only the plasma samples included in the training set are displayed. The regional FSR was calculated at a specified bin by dividing the number of fragments sized in the short range (80–150 bp) by those in the long range (151–220 bp). The profile of each sample is shown as a horizontal line. **b** Boxplot displaying the global fragment size ratio of each sample (points) across five cohorts (*x*-axis). For cancer cohorts, the samples are further grouped by cancer stage. **c** Correlation in the regional FSR profile between each sample and the healthy representative, which was composed of the binwise median values.

The genome-wide fragment size distribution demonstrated the well-known shape in which healthy controls usually exhibit the highest mode, near 167 bp, with periodic oscillations observed on the shorter end (Fig. 5a)^64^. Cancer samples are often enriched for short fragments, but the degree varies depending on the stage and the type of cancer. The short (80–150 bp) to long (151–220 bp) fragment (S/L) ratio was calculated to quantify the enrichment (Materials and Methods). Figure 5b displays the distribution of the global ratio across cohorts and cancer stages. The median ratio observed in the healthy cohort was 0.2, with an associated SD of 0.036. In the colon cancer cohort, the ratio generally increased with cancer stage; however, several outliers were observed for stage 4 samples. Liver and lung cancer also presented a moderately increasing trend by stage.

The S/L ratios were also computed at the regional level. The normalized ratio profile (“Materials and methods”) illustrates the difference among samples and cohorts (right panels in Fig. 5a). The healthy cohort demonstrated relatively synchronized profiles, but cancer cohorts exhibited increased variability. To quantify the differences between healthy and cancer samples, we calculated the correlation between the healthy median profile (constructed from the median ratio of healthy controls in each bin) and the profile of each sample (Fig. 5c). Clearly, the correlation was highest among healthy controls and tended to decrease with increasing stage in cancer patients.

Overall, we demonstrated that the fragment size ratio (FSR) profile reflects the cancer signature in cfDNA but at varying magnitudes. Interestingly, unsupervised clustering of cfDNA samples suggested only that the cancer signal could be weaker in fragmentation patterns than in methylation or copy number profiles (Supplementary Fig. 15).

### Cancer signature ensemble model for accurate cancer detection

Previous results indicated that the AMF, CNR, and FSR are all effective features for capturing cancer signals that can be simultaneously obtained from our assay. To investigate the improvement obtained by integrating these factors, we first built a classifier for each feature type and then constructed a CSE classifier using the individual classifier scores (“Materials and methods”).

**Fig. 6:**
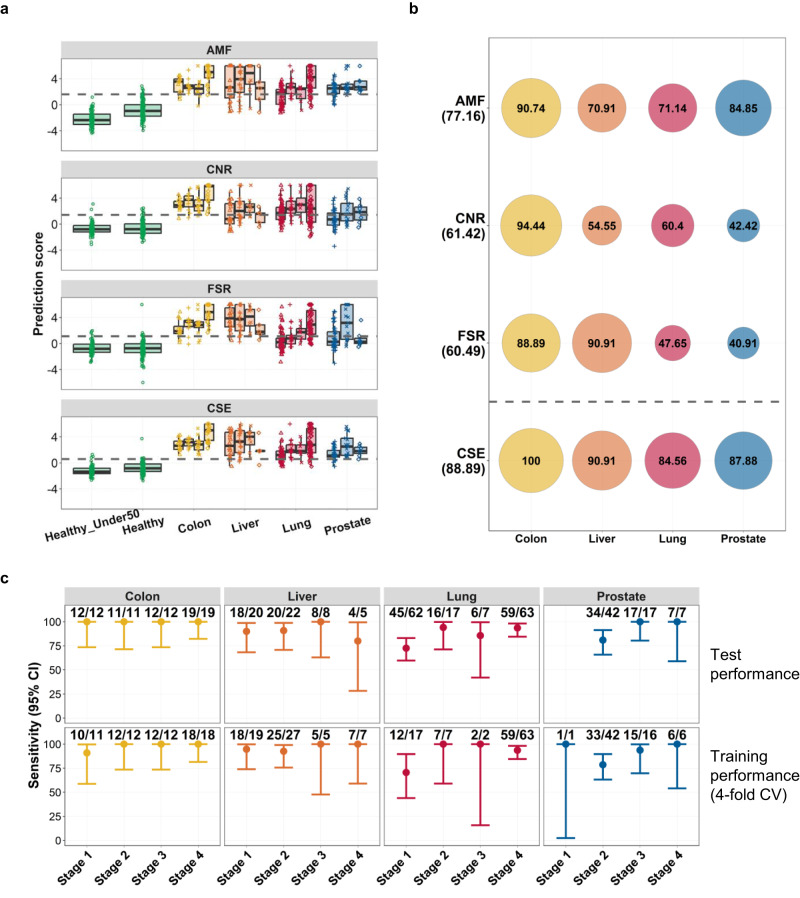
Performance of the cancer detection classifiers. **a** Boxplot displaying the prediction score of each cancer detection classifier by cohort and cancer stage. Each panel shows the score distribution of the average methylation fraction (AMF), copy number ratio (CNR), fragment size ratio (FSR), and cancer signature ensemble (CSE) classifier. The healthy samples of individuals younger than 50 years (“Healthy-under50”) are separately grouped from those of individuals older than 50 (“Healthy”). The dotted horizontal gray line indicates the cutoff determined *post hoc* to achieve >95% specificity in the test set. **b** Sensitivity computed for each cancer type (columns) by each classifier (rows). The value is shown as a text (unit in percentage) within the surrounding circle proportionally sized and color matched by cancer type. The overall sensitivity of each classifier is shown below the classifier name on the left side of the figure. **c** Sensitivity and its 95% confidence interval displayed as a vertical range bar across cancer stages (*x*-axis) for each cancer type (panel). The numbers on top of the vertical range bar denote the number of detected samples showing a cancer signature per cancer type and stage. The upper row summarizes the independent test performance, and the lower row summarizes the four-fold cross-validation training performance.

The CSE model outperformed all individual classifiers. Receiver operating characteristic curves indicated that the CSE improved the classification performance over the whole spectrum (Supplementary Fig. 16). The areas under the curve (AUCs) for the AMF, CNR, and FSR classifiers were 0.95 [confidence interval (CI) 0.94–0.96], 0.91 [CI 0.89–0.94], and 0.89 [CI 0.87–0.92], respectively, but the value was increased to 0.98 [CI 0.97–0.99] by the CSE. The score distribution from all classifiers is shown in Fig. 6a. For cancer patients, although minor, an increasing trend was observed with cancer stage, especially with the CSE. Healthy samples were evaluated separately for the groups with patients older (our target population) or younger than 50 years. For the methylation classifier, the median score in the former group was higher than that in the latter (significant at a *p* value of 0.003). This finding may not be surprising because methylation is well known to be associated with aging^65^. Such a difference was not observed for the CNR and FSR features, implying that integrating multiple features could modulate this extra signal.

When the threshold was set ensuring >95% specificity (corresponding to 95.2% [CI 90–98] using the healthy cohort aged >50 years), the sensitivity values obtained by the AMF, CNR, and FSR classifiers were 77.2% [CI 72–82], 61.4% [CI 56–67], and 60.5% [CI 55–66], respectively (Fig. 6b and Supplementary Table 3). With the CSE, the sensitivity was increased to 88.9% [CI 85–92], which is a significant improvement (*p* value < 0.0001) compared with the best individual classifier performance, which was obtained by methylation. The classification results for healthy controls younger than 50 years assured that the false-positive rate was also controlled at below 5% in the younger population; 96.4% [CI 90–99] of samples were scored as negative with the CSE (Supplementary Table 3). The sensitivity breakdown by cancer type (Fig. 6b) showed that the performance gain was more pronounced with lung and prostate cancers, for which poorer performance was usually exhibited than in colon and liver cancers. The sensitivities of individual classifiers ranged from 40% to 85%, but the ensemble model stably exhibited greater than ~85% sensitivity for both lung and prostate cancers.

When restricted to stages 1 and 2, the sensitivity was increased to 83.9% [CI 78–89] with the CSE compared with the values obtained with individual AMF, CNR, and FSR classifiers, which were 69.9% [CI 63–76], 55.4% [CI 48–63], and 47.3% [CI 40–55], respectively (Supplementary Fig. 17 and Supplementary Table 3). The performance gain again appeared to be more pronounced for lung and prostate cancers, for which individual classifiers sometimes exhibit less than 50% sensitivity.

Pairwise correlations between individual classification scores were between 0.71 and 0.77 (Supplementary Fig. 18), but the ensemble classifier achieved the best performance. This finding likely implies that the complementary nature of the three feature types enhances the stability of the classification. The sensitivity of the CSE model by cancer type and stage is summarized in Fig. 6c. The independent test performance of the CSE model was comparable to the cross-validation training performance across cancer types and stages (Fig. 6c).

Our ensemble approach combining the AMF, CNR, and FSR classifiers significantly improved cancer signal identification and robust behavior. The benefit appears to be larger for difficult cancer types with poorer performance.

### Cancer signature ensemble model for tissue of origin prediction

TOO prediction was performed on samples that were classified as suspected cancer. Similar to cancer detection using the CSE, an ensemble classifier for the TOO was constructed by averaging scores of individual classifiers based on the AMF, CNR, and FSR features (“Materials and methods”). However, a different methylation set was used for the TOO methylation classifier that included more organ-specific signals. A total of 294 cancer-suspected samples (by cancer detection using the CSE) were subject to TOO classification: 6 healthy, 54 colon cancer, 50 liver cancer, 126 lung cancer, and 58 prostate cancer samples.

**Fig. 7:**
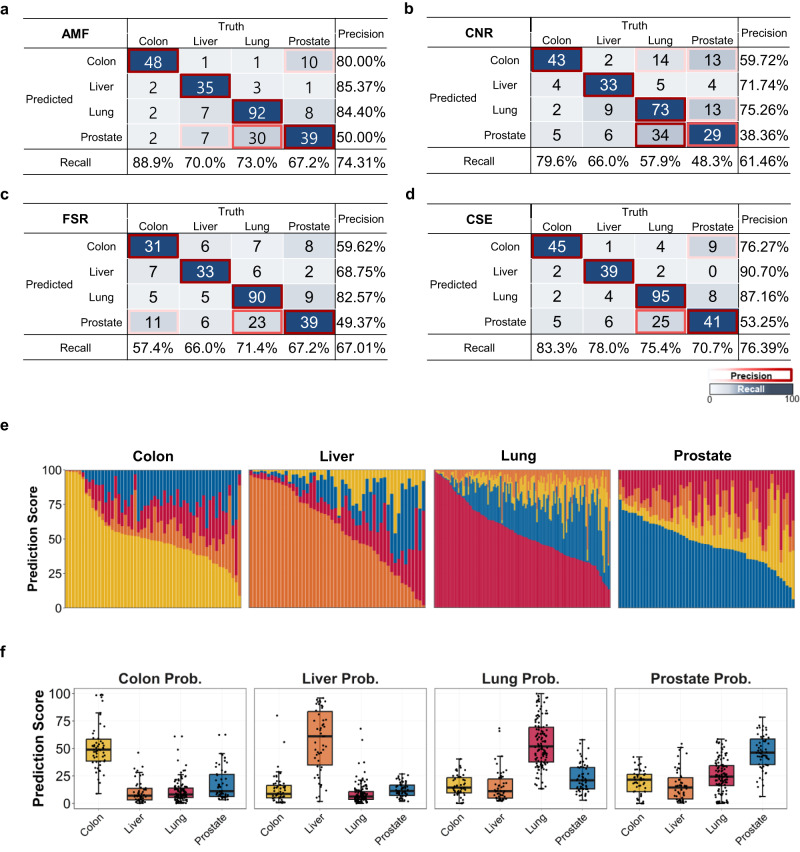
Performance of the tissue of origin classifiers. Confusion matrix summarizing the tissue of origin (TOO) classification results for the **a** average methylation fraction (AMF), **b** copy number ratio (CNR), **c** fragment size ratio (FSR), and **d** cancer signature ensemble classifier. Accuracy (bottom horizontal axis) and precision of CSE prediction (right vertical axis) among true positive participants with a known cancer signal origin. Correct TOO calls are indicated on the diagonal. **e** Stacked bar plot visualizing the conditional TOO probability (*y*-axis) of CSE decomposition in each sample (*x*-axis). The samples are grouped by cancer type. Samples on the x-axis are ordered by high conditional probability of their own cancer type. **f** Boxplot showing each organ-supporting probability of CSE (titled in each panel) across four cancer cohorts (*x*-axis).

The prediction accuracy of cancer samples by the AMF, CNR, and FSR classifiers was 74.3% [CI 69–79], 61.5% [CI 56–67], and 67.0% [CI 61–72], respectively (Fig. 7a–c). The methylation feature achieved the best performance overall and for individual recall and precision values computed by cancer type. The CNR and FSR exhibited worse performance, but the CNR had a slightly better predictive value for colon cancer, and the FSR had better predictive value for lung and prostate cancer. The CSE constructed for TOO that integrated all features achieved an improved accuracy of 76.4% [CI 71–81] (Fig. 7d). Although not statistically significant, the CSE performed better than the methylation classifier in most aspects, with recall values of 83.3%, 78.0%, 75.4%, and 70.7% for colon, liver, lung, and prostate cancers compared with values of 88.9%, 70.0%, 73.0%, and 67.2%, respectively, by methylation. Similarly, a minor gain was observed in precision.

The conditional probability decomposition, which showed the supporting origin for each sample (Fig. 7e), revealed the associated confidence for classification and competing organ types. For example, 75% of lung samples retained the correct origin according to the maximal probability, but prostate origin was repeatedly found to be the second highest candidate. When the probabilities were organized by supporting origin (Fig. 7f), the colon and liver cohorts demonstrated an exclusively high score distribution by true origin, but the lung cohort had a less specific distribution, and the prostate cohort presented a minimally specific distribution. Many lung samples had competitive scores compared with prostate samples, resulting in nearly 50% precision. This suggests that the CSE model was relatively weak in distinguishing between lung and prostate cancers. In contrast, colon and liver cancers presented strong unique signatures (Supplementary Fig. 19).

Among the individual feature types, methylation exhibited the best TOO performance compared with the copy number and fragmentomic patterns. However, integration of all features led to a further improvement in accuracy.

## Discussion

In this study, we introduced AlphaLiquid^®^ Screening, a cfDNA NGS platform that enables integrative analysis of genome-wide methylation, copy number, and fragmentomic patterns. Nearly 1000 cfDNA and 250 tissue samples were analyzed, allowing us to thoroughly demonstrate the utility of each feature and, more importantly, the power of using an all-in-one assay for the early detection of multiple cancers. When evaluated on an independent test set, our CSE classifier (combining the three features) achieved 88.9% sensitivity at 95.2% specificity for cancer detection, which is more than 10% higher than that of any other classifier built on individual features. Regarding localization of the TOO, we found that a methylation classifier built on a selected marker set enriching tumor-specific signals exhibited the best performance. Nonetheless, the genome-wide fragment size profiles and copy number patterns also carried modest signals; thus, another ensemble classifier was built to benefit the simultaneous availability of all features.

Direct comparisons of our classification performance with those of other studies in the literature are difficult because the cancer type and stage or clinical characteristics would differ. Additionally, many other studies involve similar individual feature types that are differently engineered^20,32,33,37,39,66–70^. However, the relative contribution of each feature type in identifying cancer signals would likely be maintained. Indeed, we found similar conclusions in a recent benchmark study^40^. The genome-wide methylation signature is the most promising model for tracing the tissue origin as an individual feature type, and a panfeature model combining methylation, copy number, fragmentomic, and mutation patterns from multiple assays, including whole-genome bisulfite sequencing at ~30×, whole-genome sequencing at ~30×, and targeted deep sequencing at ~60,000×, achieved the best performance for cancer detection^40^. We note that unlike this recent study that utilized different datasets, our platform was able to capture all of the features from a dataset generated by a single experimental assay.

Our study has several limitations. First, we examined only portions of cancer-associated features that can be obtained from cfDNA sequencing. For example, microbial abundance, fragmentomic endpoint distribution, or motif patterns have also been studied regarding cancer association^71,72^. Second, the CSE models presented were constructed using samples from the Korean population and included only four cancer types, which do not represent the cancer prevalence in Korea or elsewhere and cannot reflect ethnic diversity. Third, limited demographic and clinical characteristics were available; thus, the exclusion criteria were based only on samples with available information.

Maximum utility of screening platforms is achieved when they are applied to detect most frequently occurring cancers. With the accumulation of more cancer types in the models, we intend to commercialize our platform. A draft report page presenting the results of the CSE models for cancer detection and tissue of origin findings is shown in Supplementary Fig. 20. Additional efforts will include broadening the ethical spectrum through global collaborations. In the meantime, our platform has maximal potential to be adapted to address other biological or clinical questions, such as searching for biomarkers or classifying contrasted groups. Because the availability of features is not restricted to biased regions selected for cancer detection, a flexible analysis could be performed for nearly any purpose. Continuous accumulation of data from our assay along with associated clinical information will provide additional value to advance the liquid biopsy field in precision oncology as comprehensive profiling of epigenetic and genetic signatures can be easily revisited.

### Supplementary information

Supplementary data

Supplementary tables

## Data Availability

Raw data for this study were generated at IMBdx, Inc. Derived data supporting the findings of this study are available from the corresponding author upon request.
