# Supplementary material for: Cancer signature ensemble integrating cfDNA methylation, copy number, and fragmentation facilitates multi-cancer early detection
Source: Exp Mol Med. 2023 Nov 1;55(11):2445–60. doi: 10.1038/s12276-023-01119-5 (PMC10689759; doi:10.1038/s12276-023-01119-5)
Supplement: Supplementary file 1 — Supplementary data [file 12276_2023_1119_MOESM1_ESM.pdf]

Supplementary Data

Cancer signature ensemble integrating cfDNA methylation, copy number, and fragmentation facilitates multi-cancer early detection

Su Yeon Kim *et al.*

* Corresponding author. Email: duheebang@yonsei.ac.kr (D.B.); kimty@snu.ac.kr (T.-Y.K.)

This PDF file includes:

Supplementary Figs. 1 to 20

Other Supplementary Data for this manuscript include the following:

Supplementary Tables 1 to 3

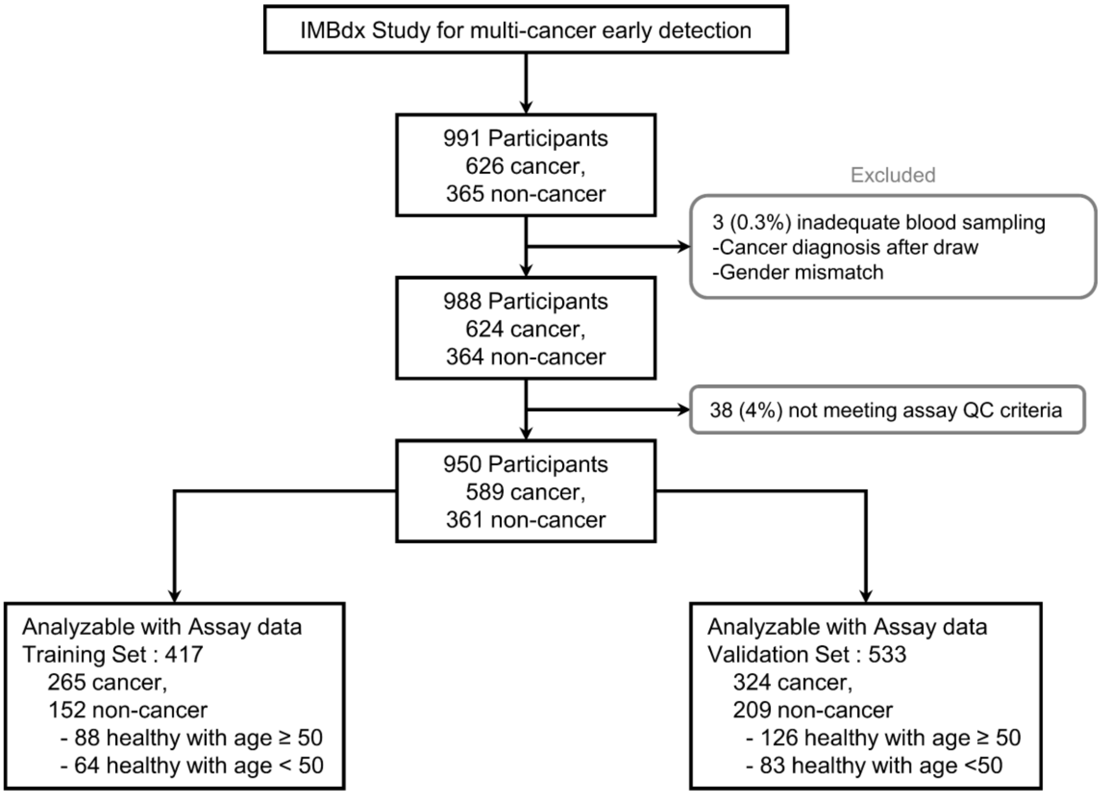

Supplementary Fig. 1. AlphaLiquid® Screening study.

Data from 991 participants were collected, including 626 cancer patients and 365 healthy controls. Three participants were excluded due to inadequate blood sampling and an additional 38 samples were filtered out by assay quality control (QC) criteria. The remaining participants were divided into training (n=417) and test (n=533) sets for machine learning.

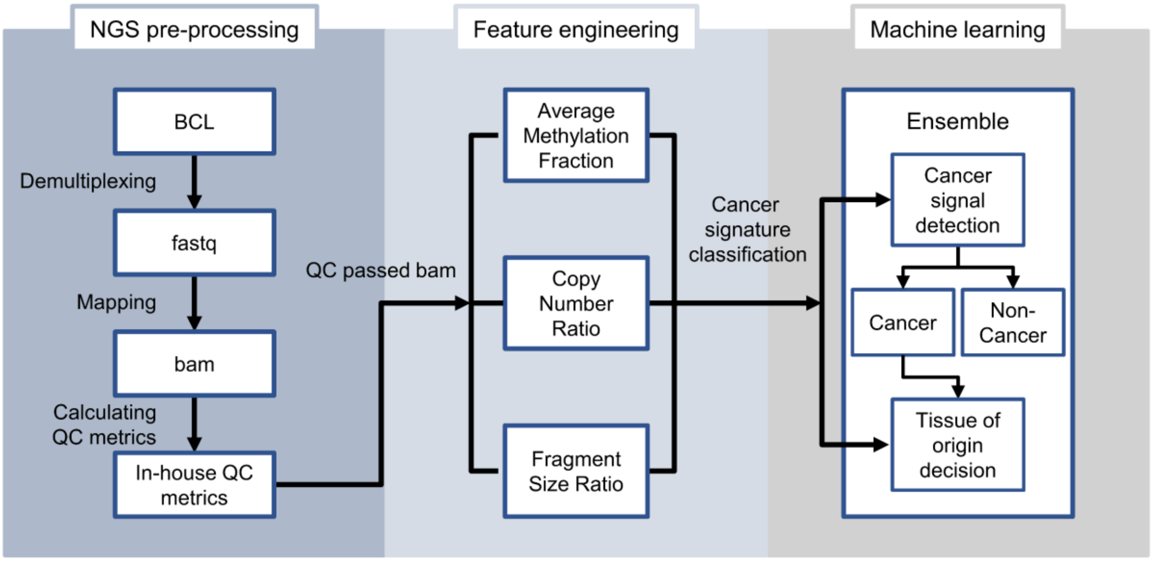

Supplementary Fig. 2. AlphaLiquid® Screening analysis workflow.
The workflow consisted of three procedures: next-generation sequencing (NGS) pre-processing, feature engineering, and machine learning application. NGS pre-processing started with demultiplexing, converting a bcl file to a fastq file, and then performing read mapping onto the GRCh37 reference genome creating a bam file. Subsequently, in-house quality control (QC) metrics were calculated. Next, the average methylation fraction, copy number ratio, and fragment size ratio features were extracted from the bam file and fed to the cancer signature ensemble model for cancer signal detection and to search for the tissue of origin.

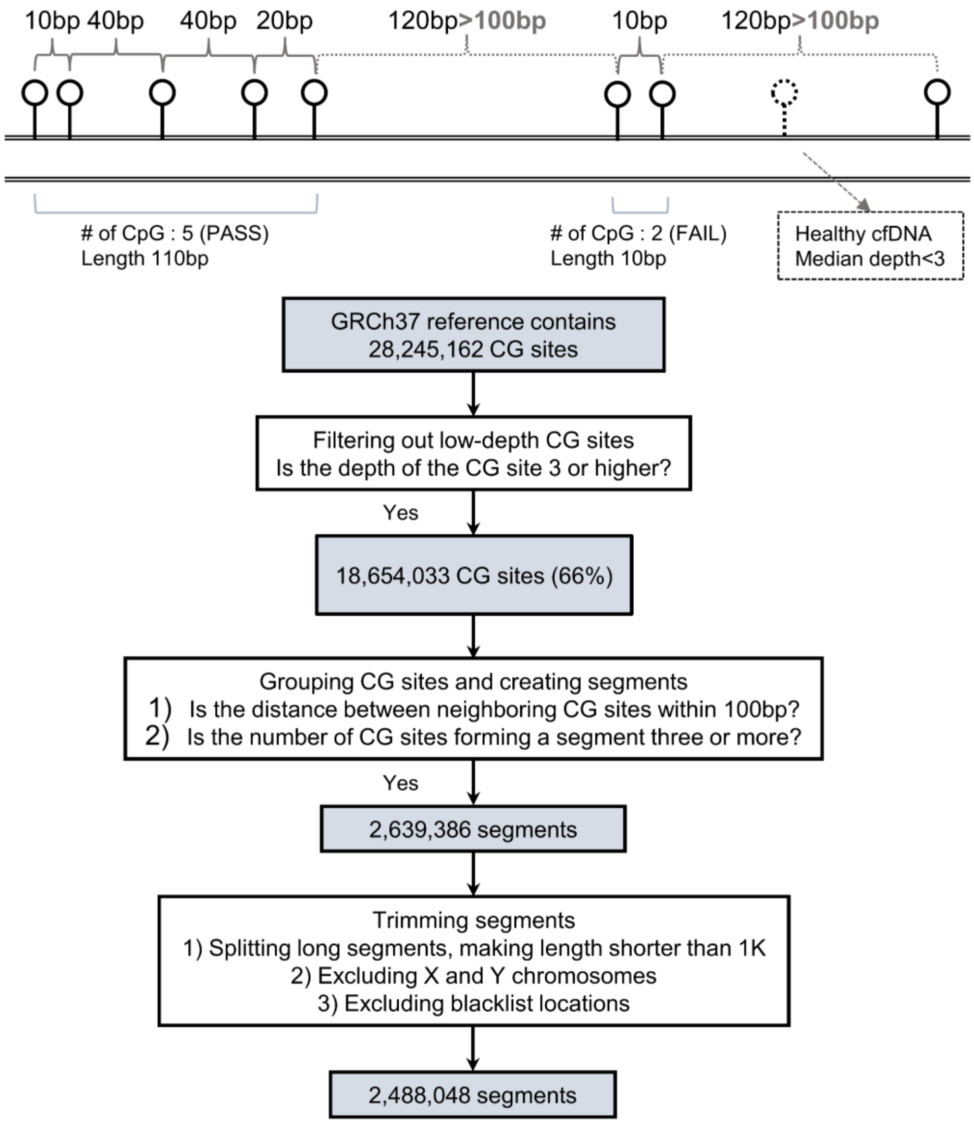

Supplementary Fig. 3. Definition of methylation regions analyzed in this study.
Starting from the ~30 million CpG sites in the GRCh37 reference genome, 2,488,048 methylation regions were obtained for downstream analyses following the steps described in the flow chart.

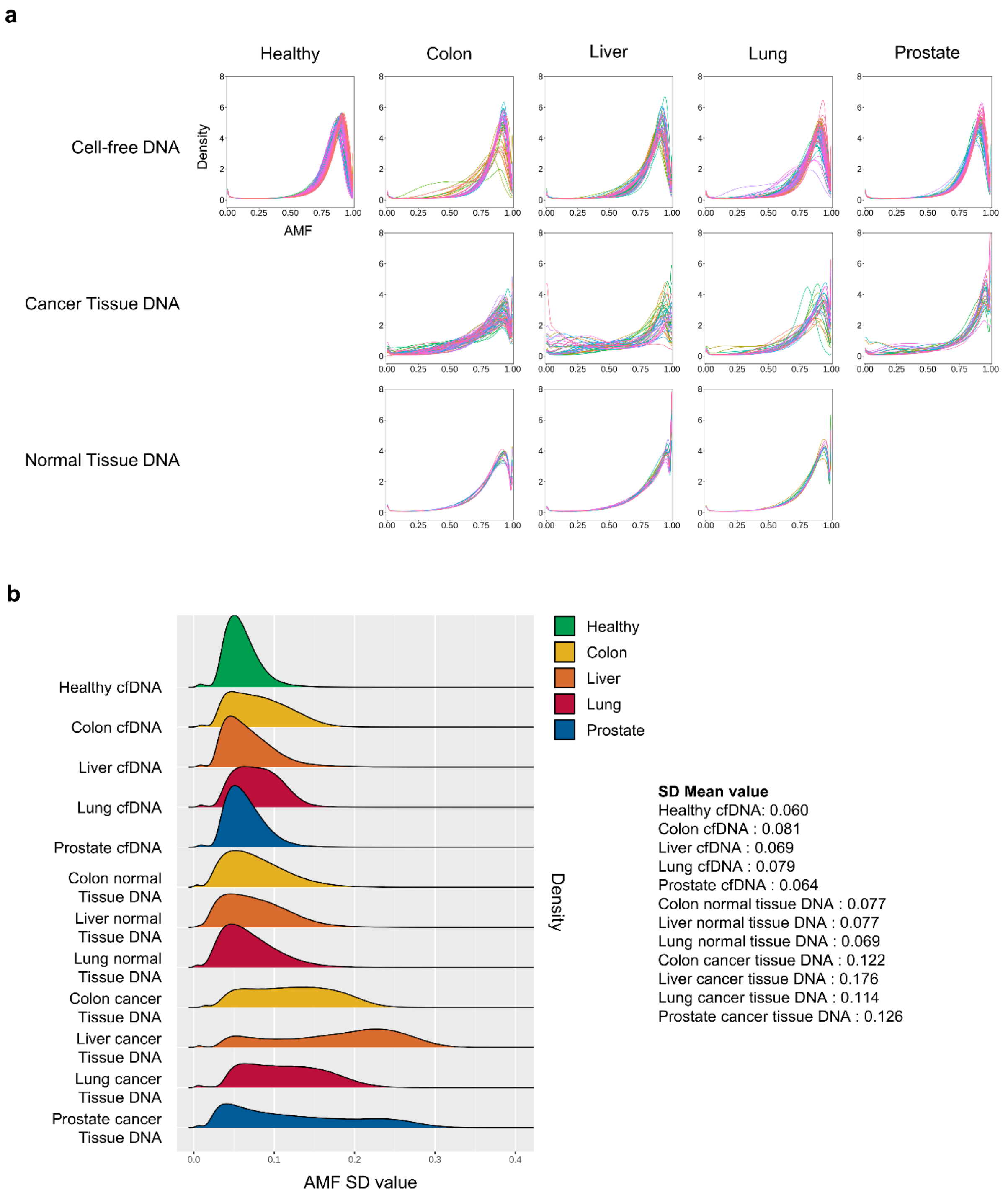

Supplementary Fig. 4. Genome-wide distribution of the regional methylation level. **(a)** Density plot of the average methylation fraction (AMF) collected from ~2.4 million methylation regions per each sample (colored lines). Samples were grouped by sample type (row) and cohort type (column). **(b)** Genome-wide distribution of the standard deviation (SD) of the AMF value calculated at each methylation region. The mean of the SD of each group is listed on the right side.

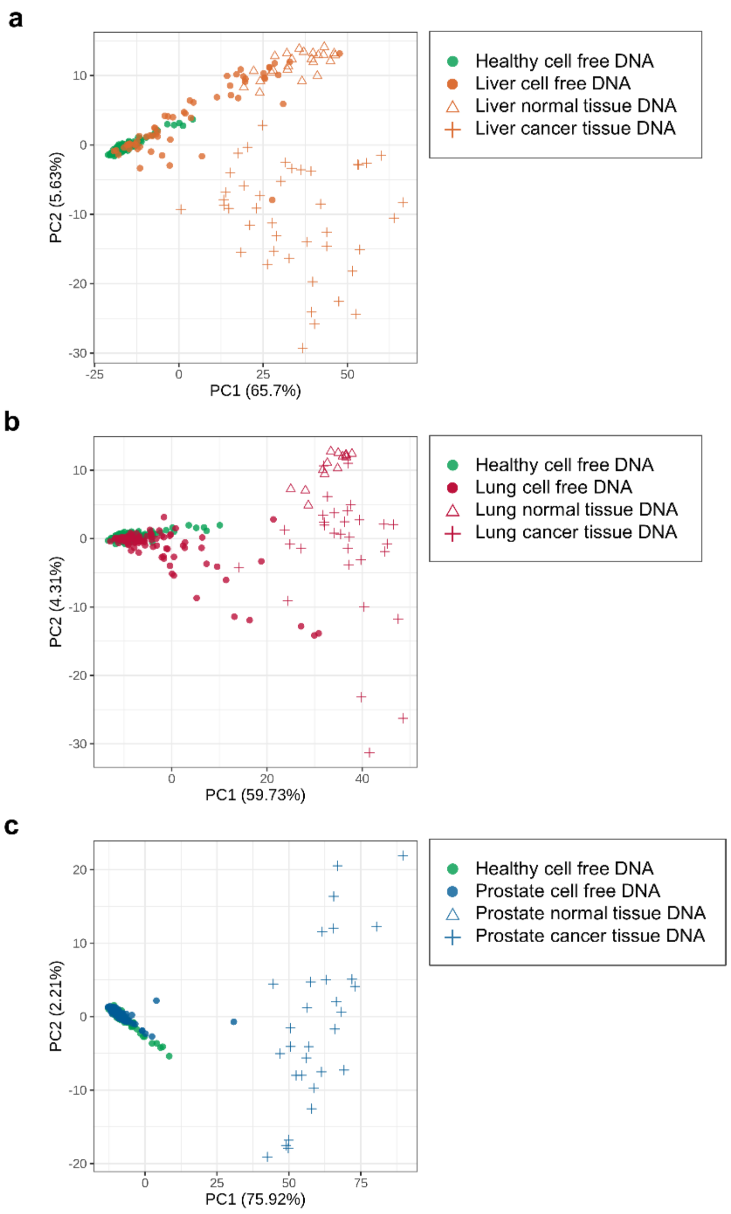

Supplementary Fig. 5. Variation in the methylation level by sample type.

Principal component analysis was performed on healthy cfDNA samples (green-filled circles) and all sample types from hepatocellular carcinoma **(a)**, lung cancer **(b),** and prostate cancer **(c)**. The average methylation fractions of ~67,000 regions with low methylation levels in the healthy training set were used for the analysis.

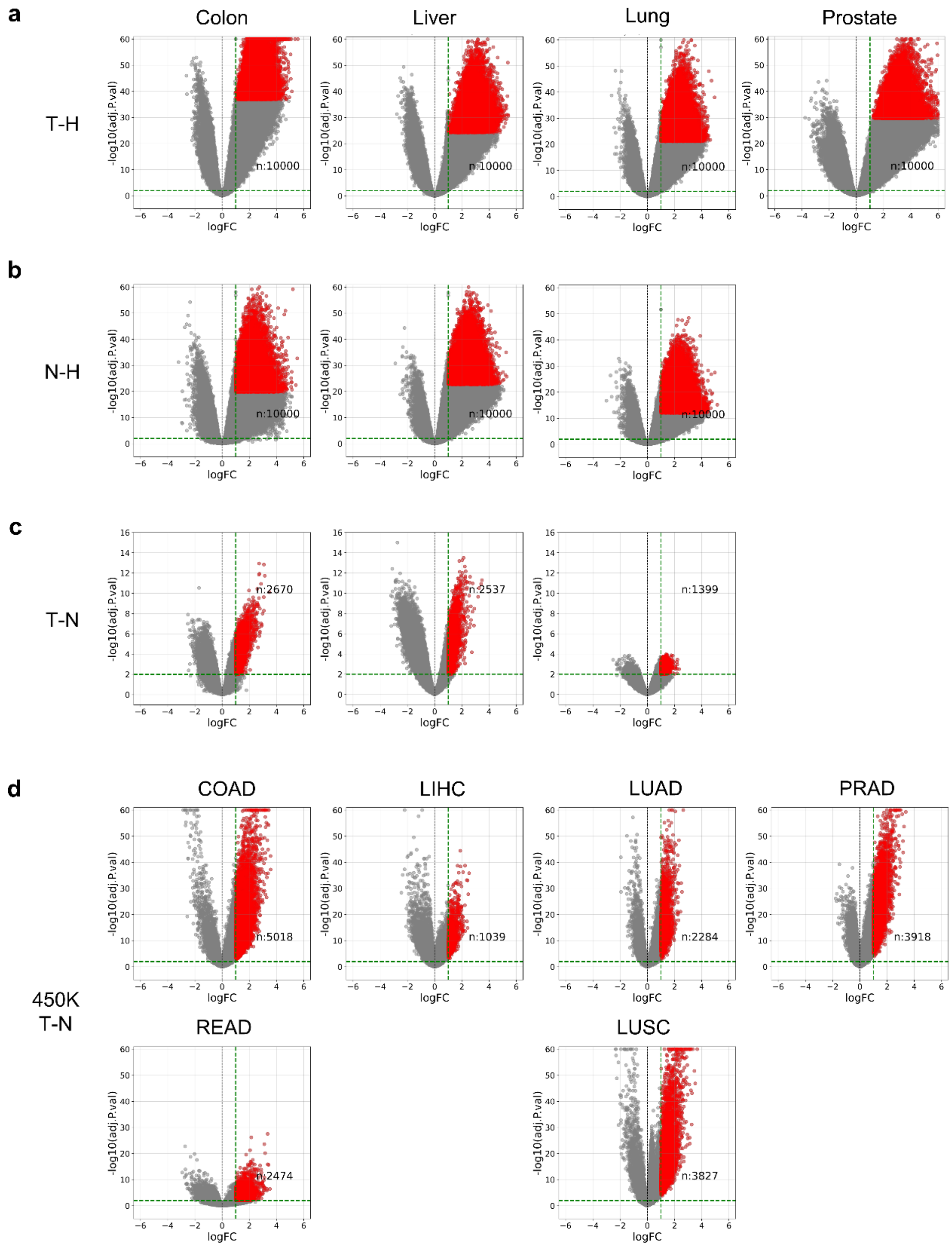

Supplementary Fig. 6. Volcano plots indicating differentially methylated markers among regions with low methylation in the healthy cohort.

Differential methylation analyses were carried out using sequencing data and The Cancer Genome Atlas 450K data for markers in the 'healthy-unmethylated' regions (for details, see Materials and Methods). For the sequencing data, comparisons were performed between **(a)** cancer tissue and healthy cfDNA ('T-H'), **(b)** normal tissue and healthy cell-free DNA ('N-H'), and **(c)** cancer tissue and normal tissue ('T-N'). For the 450K data **(d)**, comparisons between tumor and normal tissues ('T-N') were performed using the related cancer cohorts.

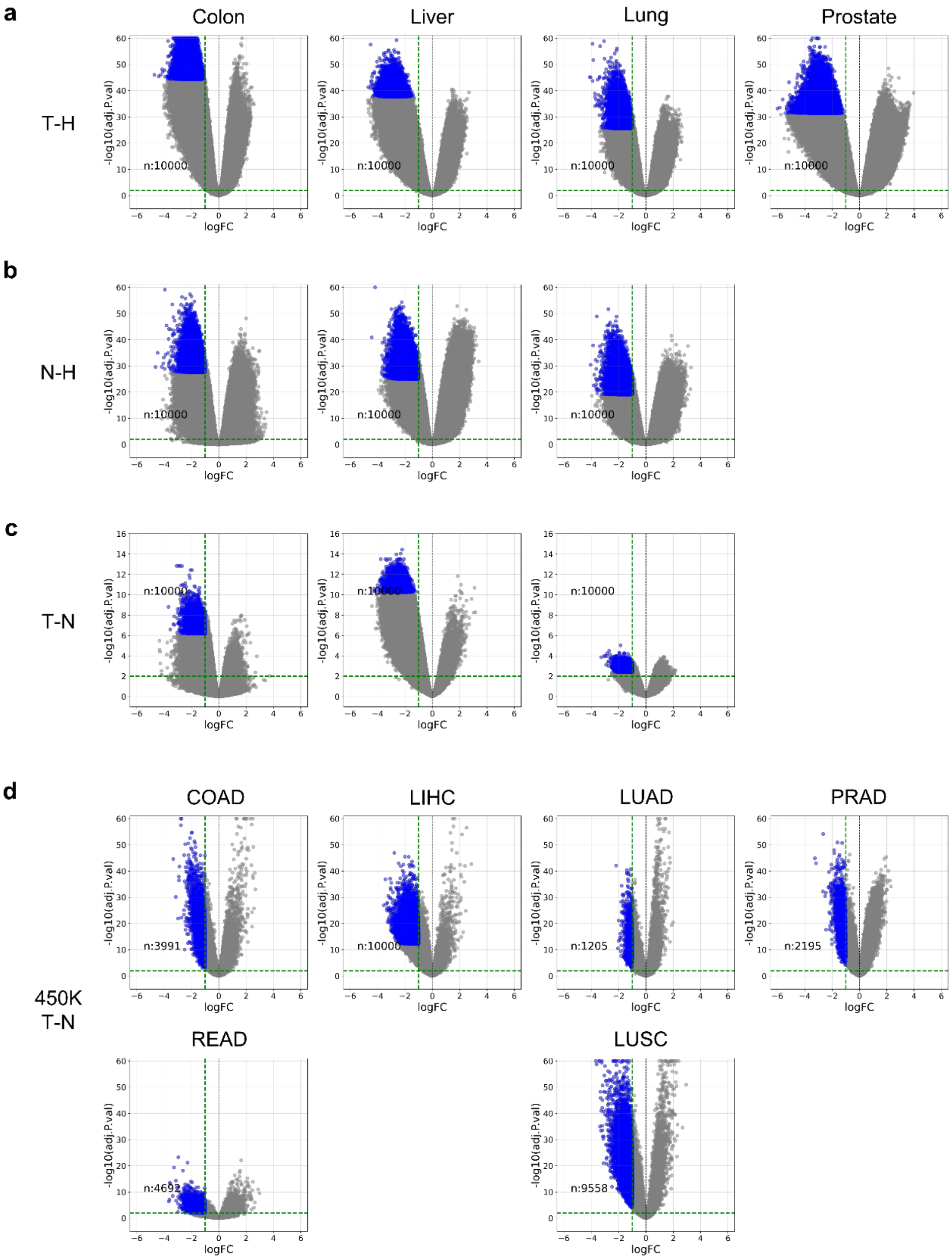

Supplementary Fig. 7. Volcano plots indicating differentially methylated markers among methylated regions in the healthy cohort.

Differential methylation analyses were carried out using sequencing data and The Cancer Genome Atlas 450K data for markers in the 'healthy-methylated' regions (for details, see Materials and Methods). Other details are similar to those in Supplementary Fig. 6.

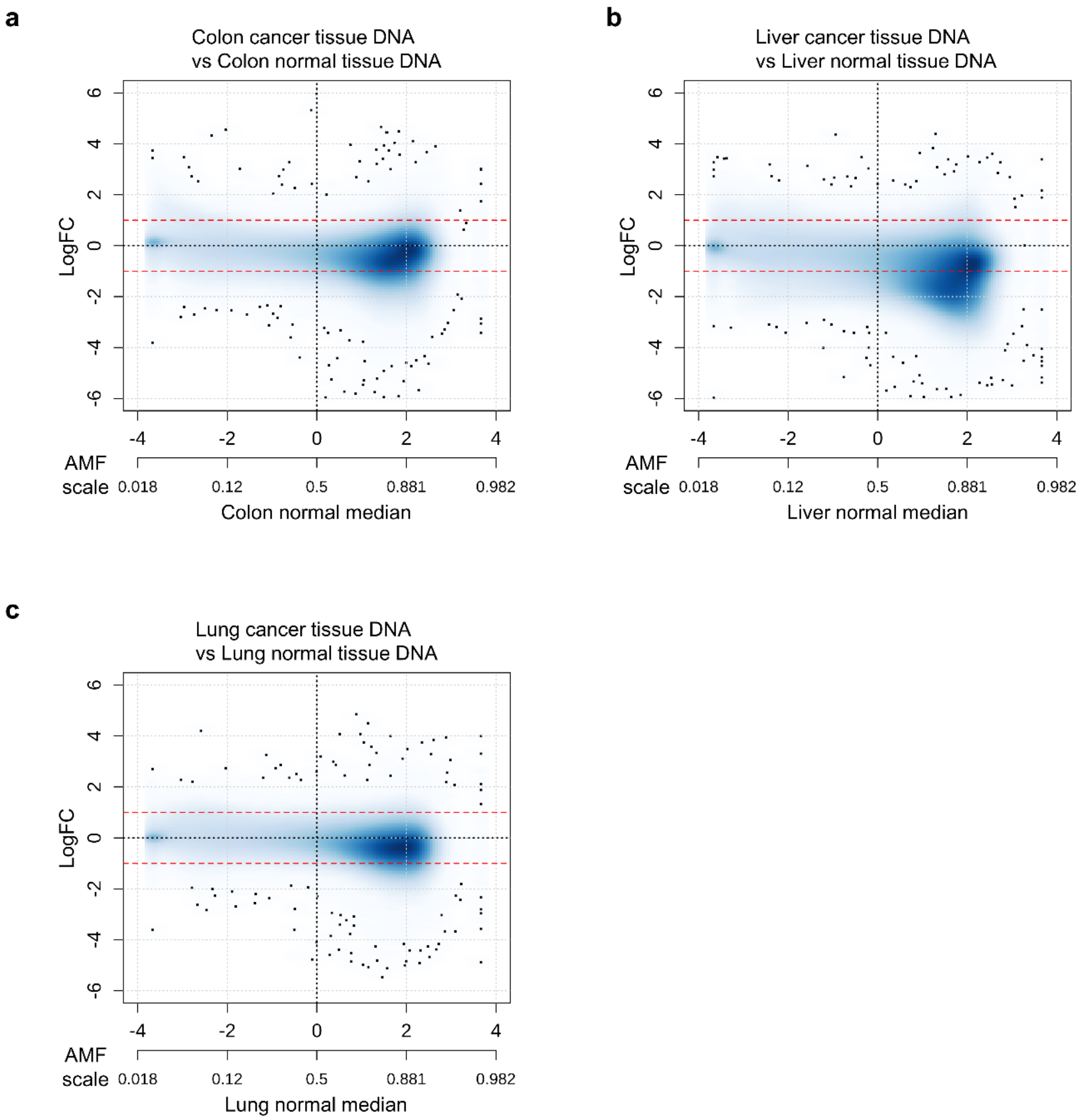

Supplementary Fig. 8. Magnitude of differential methylation levels between cancer and normal tissues.

For each colon **(a)**, liver **(b)**, and lung **(c)** cancer type, a smoothed scatter plot shows the log fold change according to the differential methylation analysis between the cancer tissues and the normal tissues, along with the median methylation level in the normal cohort.

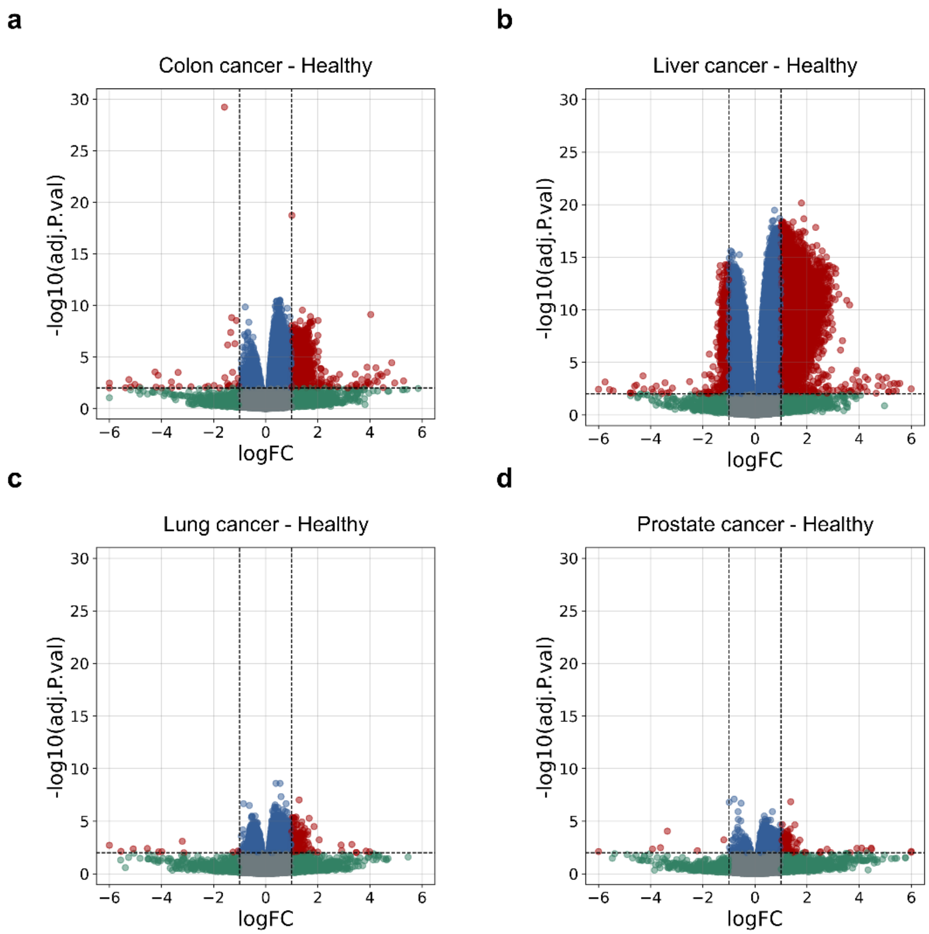

Supplementary Fig. 9. Differential methylation analysis using cell-free DNA (cfDNA) only. For each colon **(a)**, liver **(b)**, lung **(c)**, and prostate **(d)** cancer type, a differential methylation analysis was conducted comparing the associated cancer cfDNA samples with the healthy cfDNAs. Each volcano plot demonstrates the −log10 (false discovery rate-adjusted *p*-value) against the log fold change.

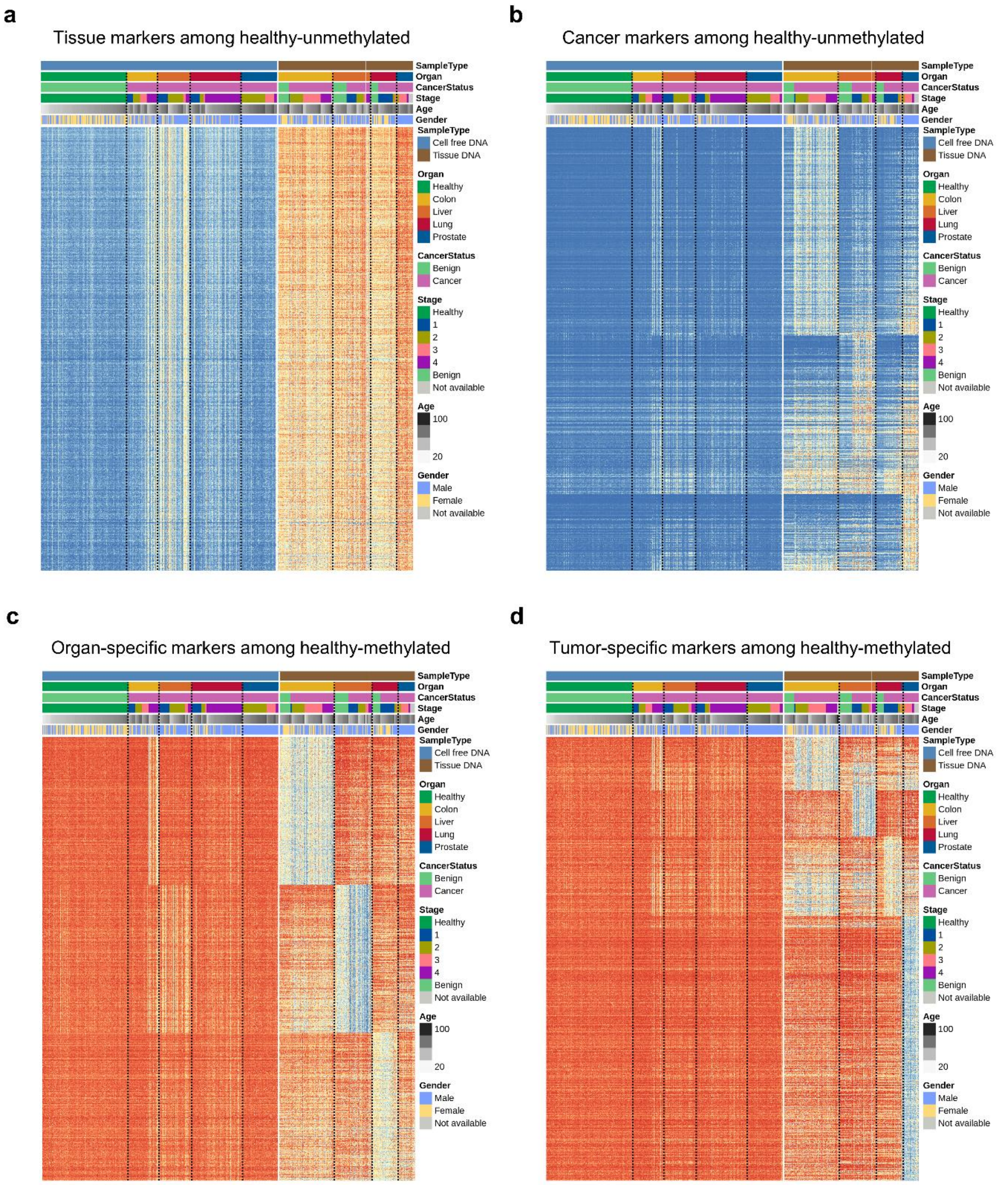

Supplementary Fig. 10. Heatmap visualizing methylation markers that reveal cancer signatures or tissue signatures.

(a) and **(b)** Differentially methylated markers between cell-free DNA and tissues (a) and between normal tissue and cancer tissues (b) among those in the 'healthy-unmethylated' regions. **(c)** and **(d)** Differentially methylated markers across organ-specific tissues (c) and between normal tissue and cancer tissue within each organ type (d) among those in the 'healthy-methylated' regions. For more details on marker selection, see Materials and Methods. The graphical details are similar to those of Fig. 3c.

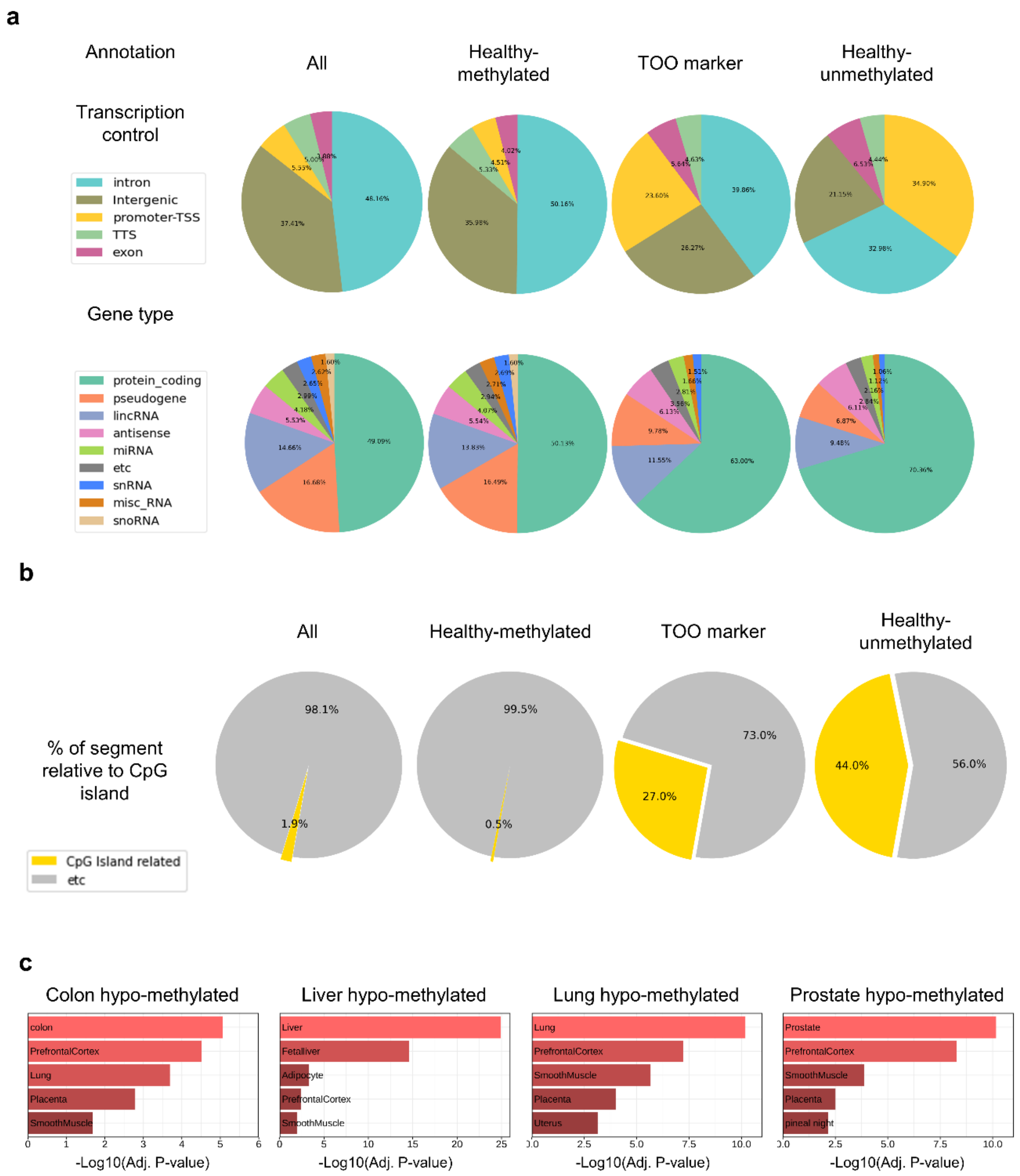

Supplementary Fig. 11. Characterization of biological functions of the methylation markers used for cancer detection and localization.

(a) Transcriptional (upper) or gene type (lower) categories were annotated and are shown as pie charts for all ~2.5 million methylation regions (‘All’), ‘healthy-methylated’ regions, regions used for tissue of origin classification, and ‘healthy-unmethylated’ regions. For details of the region definition, see Materials and Methods. **(b)** Percentage of overlapping base pairs with CpG islands for the four regions analyzed in **(a)**. **(c)** Gene set analysis of the ‘cell type’ category for the cancer-hypo markers specific to each organ (y-axis).

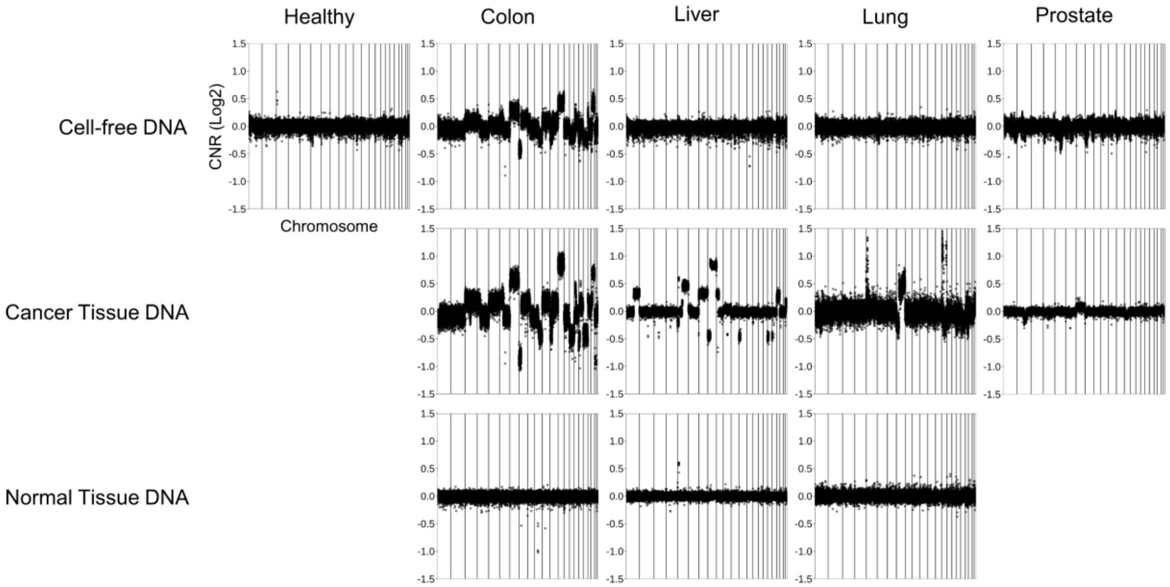

Supplementary Fig. 12. Genome-wide copy number patterns at the individual sample level. The copy number ratio (CNR) profile is shown along the genomic coordinates for cell-free DNA (cfDNA) or tissue samples. Each black point indicates the log2-transformed CNR value computed for a 100-kb bin. The gray vertical lines separate chromosomes. For colon, liver, and lung samples, the cfDNA, cancer tissue DNA, and normal tissue DNA samples were obtained from a matched donor.

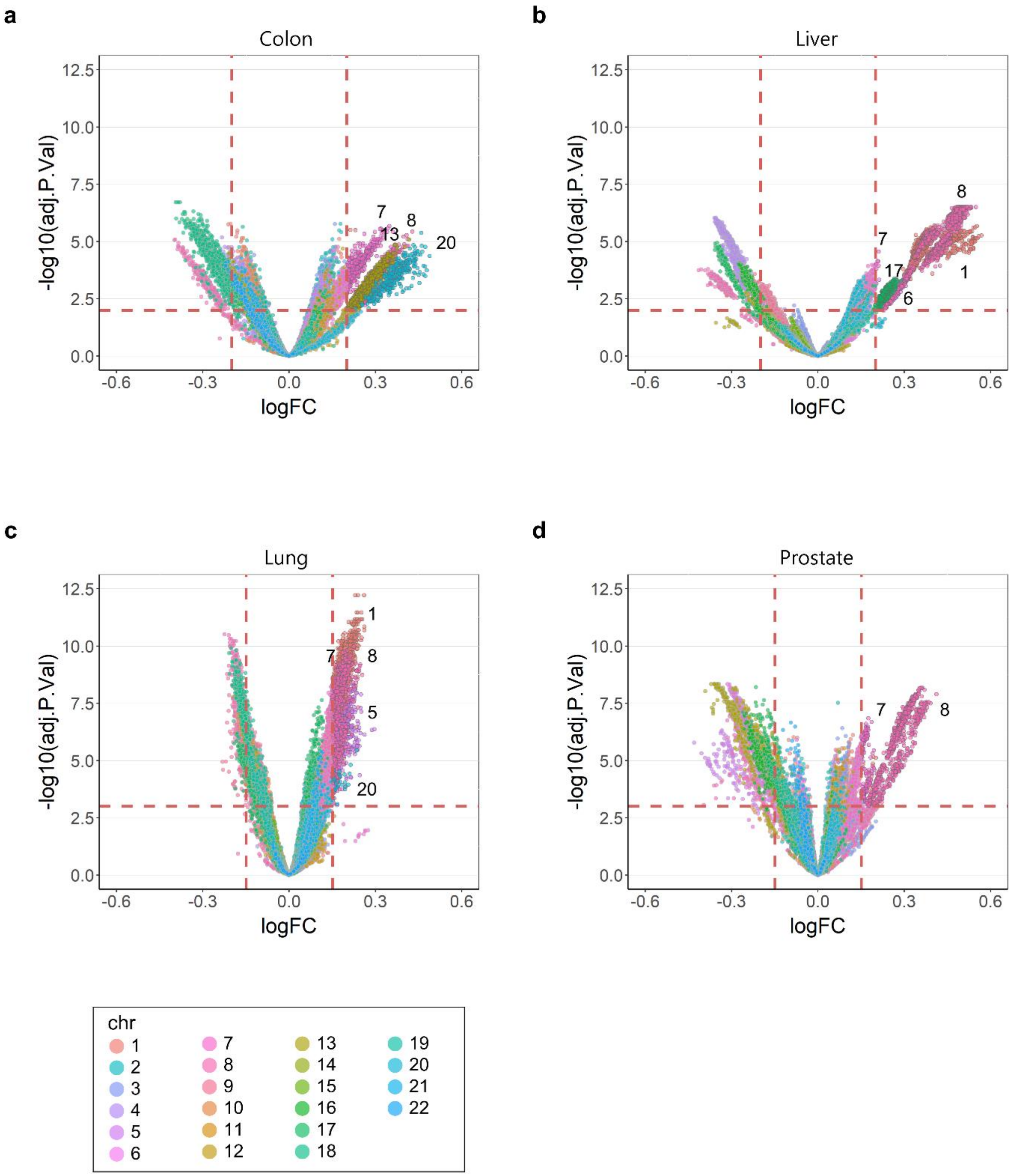

Supplementary Fig. 13. Differential copy number ratio analysis between cancer tissues and normal tissues using whole-genome methylation sequencing data.

For each colon **(a)**, liver **(b)**, lung **(c)**, and prostate **(d)** cancer, a volcano plot shows the results of differential copy number ratio (CNR) analysis (performed in log2 scale) comparing cancer tissues with normal tissues. Each CNR value calculated per 100-kb bin (one point) is colored according to the associated chromosome. Because large copy number events are often much longer than 100 kb, clusters of neighboring bins were observed. The chromosome numbers of repeated copy number gain events are indicated with black text.

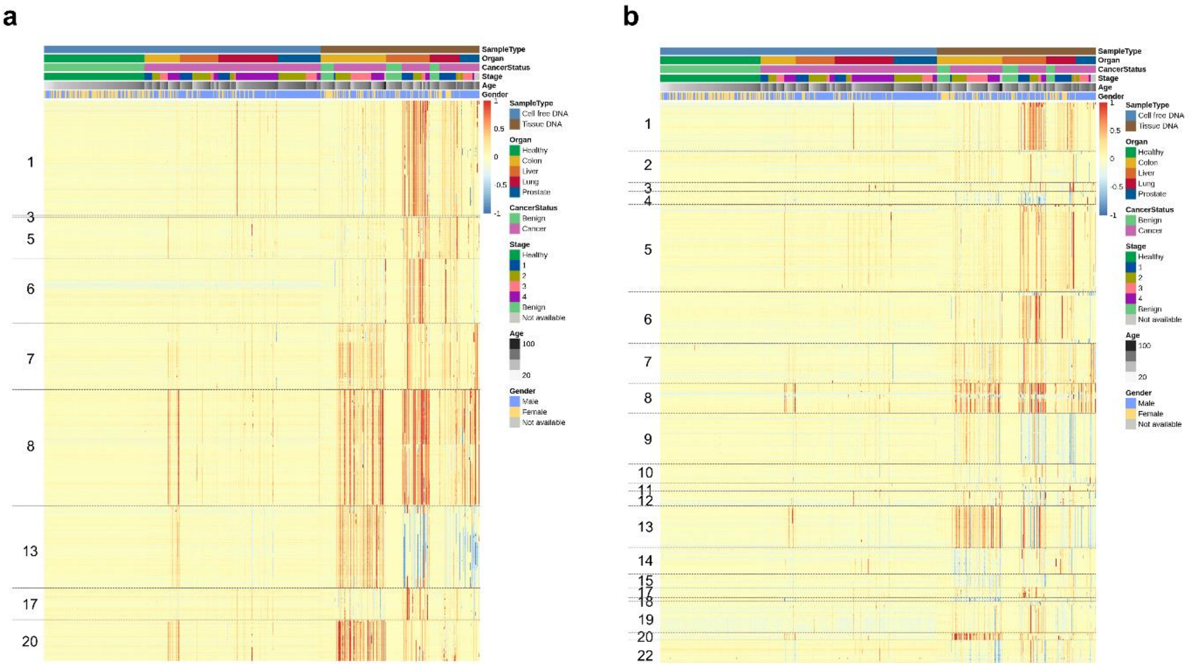

Supplementary Fig. 14. Heatmap visualizing log2-scale copy number ratio (CNR) values of our data for the copy number gain regions found by whole-genome methylation sequencing (WGMS) data and by a pan-cancer study of The Cancer Genome Atlas (TCGA).

The heatmap shows all training cell-free DNA and tissue samples for **(a)** the regions that showed a significant increase in the CNR values in our WGMS cancer tissues compared with normal tissues by any cancer type and **(b)** the regions that were reported by a pan-cancer study of TCGA as having a frequent copy number gain event. The log2-scale CNR value is plotted for each sample (columns) and genomic bin (rows). Sample annotations are shown on the upper part, similar to those in Fig. 3c, and the horizontal gray lines indicate separate chromosomes.

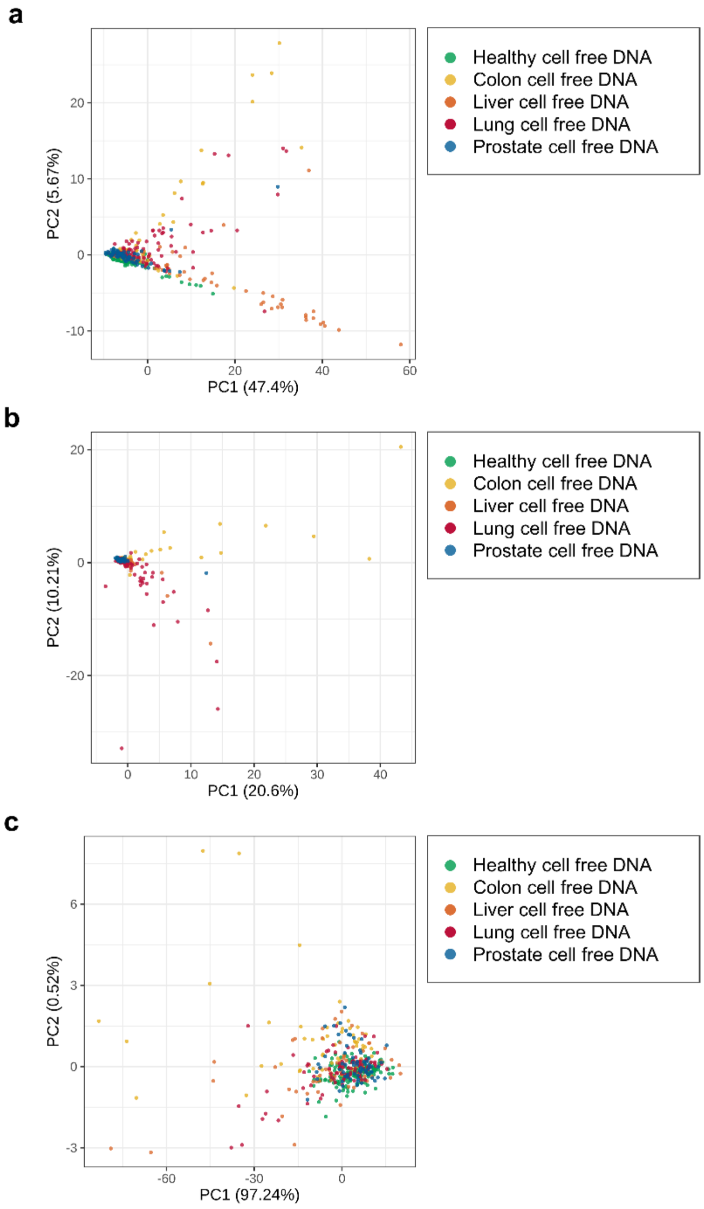

Supplementary Fig. 15. Unsupervised clustering analysis using cell-free DNA (cfDNA) samples only.

Principal component analysis of all cfDNA samples using the average methylated fraction (AMF) **(a)**, copy number ratio (CNR) **(b)**, and fragment size ratio (FSR) **(c)** features. For the AMF, ~67,000 regions with low methylation levels in the healthy training set were used. For the CNR and FSR, values were computed over non-overlapping 100-kb bins.

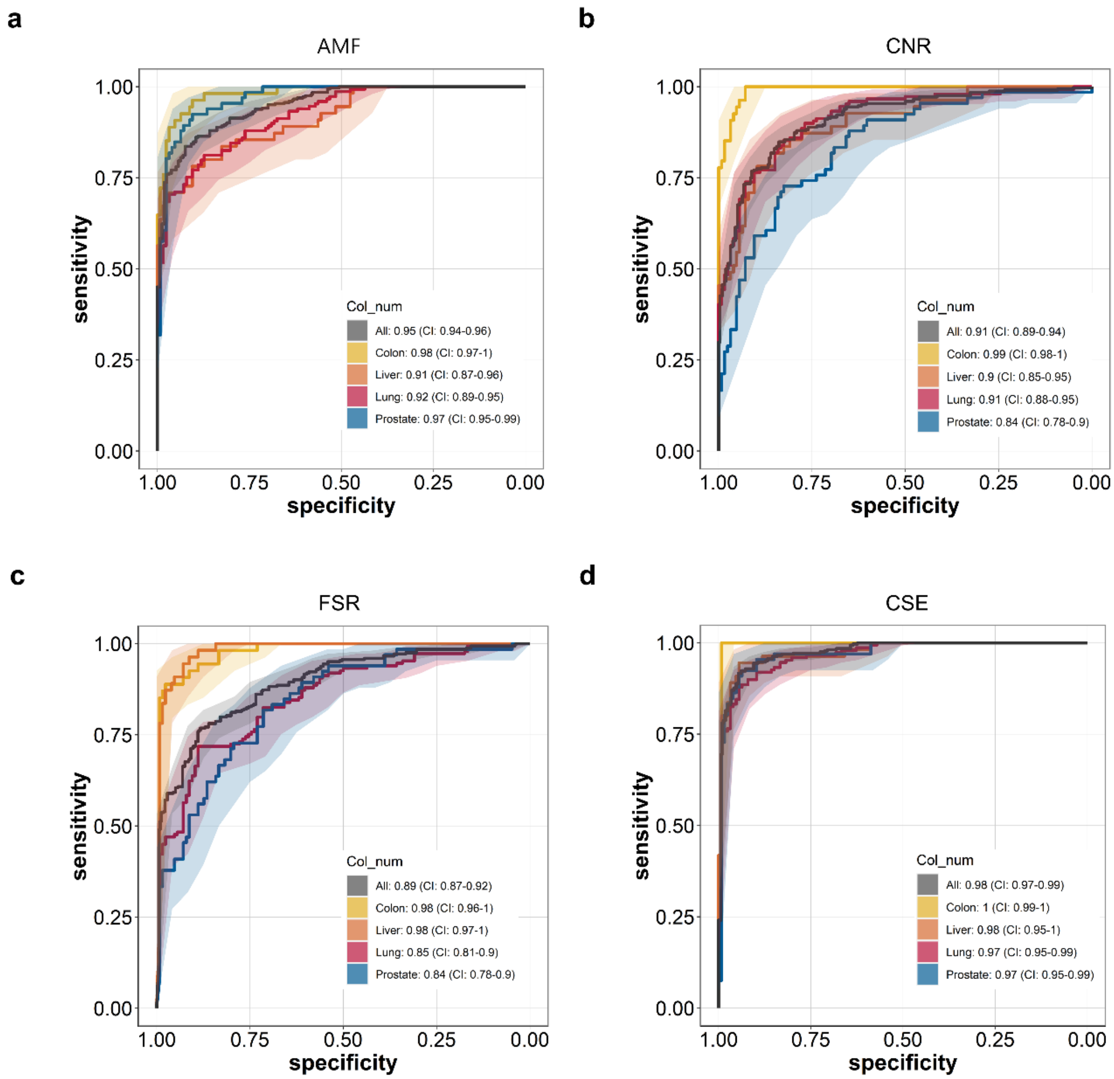

Supplementary Fig. 16. Receiver operating characteristic (ROC) curves for cancer detection classifiers.

ROC curves are shown for each cancer detection classifier, including the average methylated fraction (AMF) **(a)**, copy number ratio (CNR) **(b)**, fragment size ratio (FSR) **(c)**, and cancer signature ensemble (CSE) **(d)**, computed using all samples (black line) or including one cancer type at a time with healthy controls (colored line). The area under the curve and 95% confidence interval (CI) are shown in the lower right part of each panel.

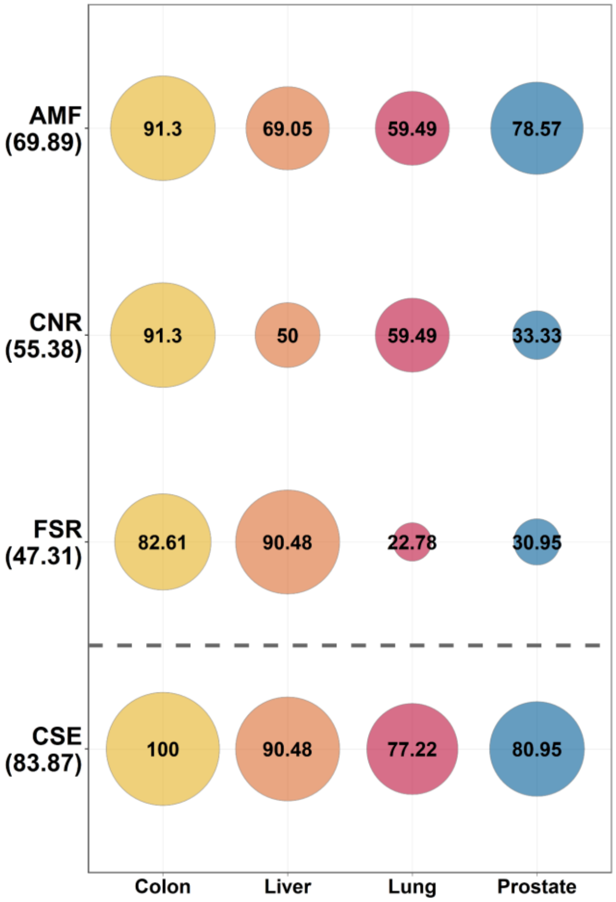

Supplementary Fig. 17. Sensitivity of cancer detection classifiers at 95.2% specificity for stage 1 and 2 cancer patients.

The sensitivity for stage 1 and stage 2 cancer patients was calculated for each cancer type (columns) by each classifier (rows). The other graphical details are similar to those in Fig. 6.

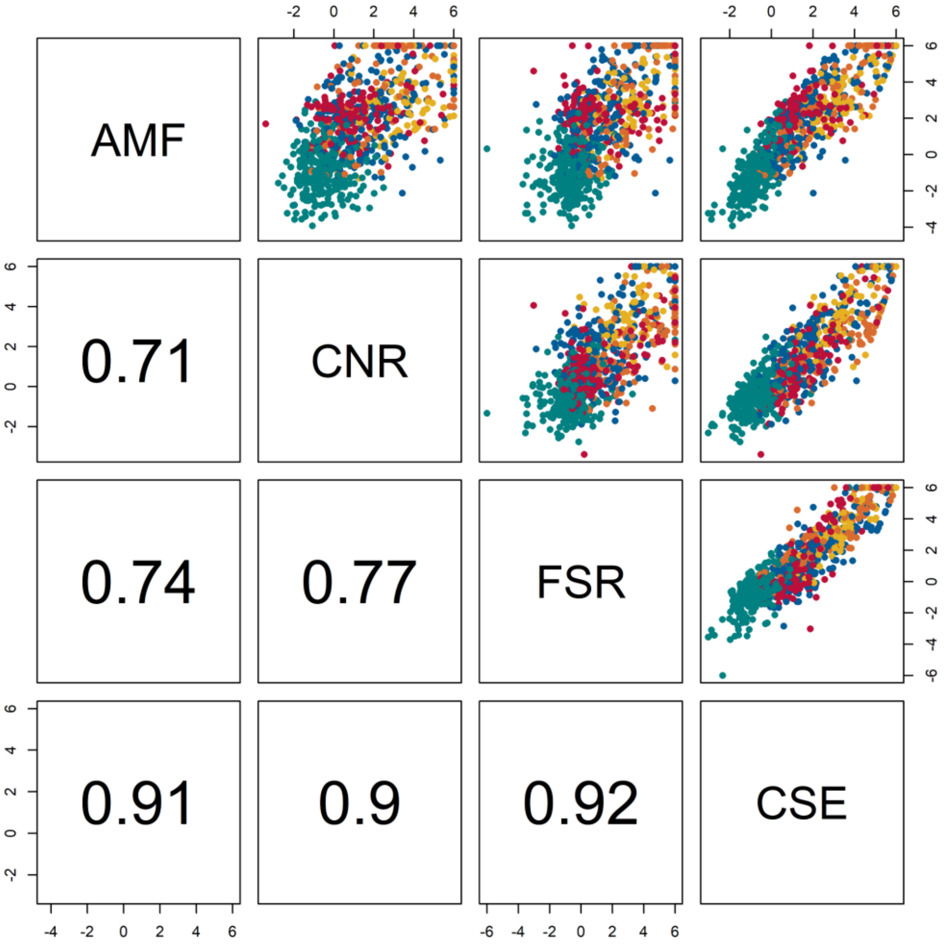

Supplementary Fig. 18. Correlation among the cancer detection classifier scores.

A pairwise correlation plot between cancer detection classifiers was constructed. The classifier name is shown in diagonal blocks; the upper part includes the scatterplot, and the lower part shows the Pearson correlation coefficient. Each sample is colored according to the cohort type: green, healthy; yellow, colon; orange, liver; red, lung; and blue, prostate cancer.

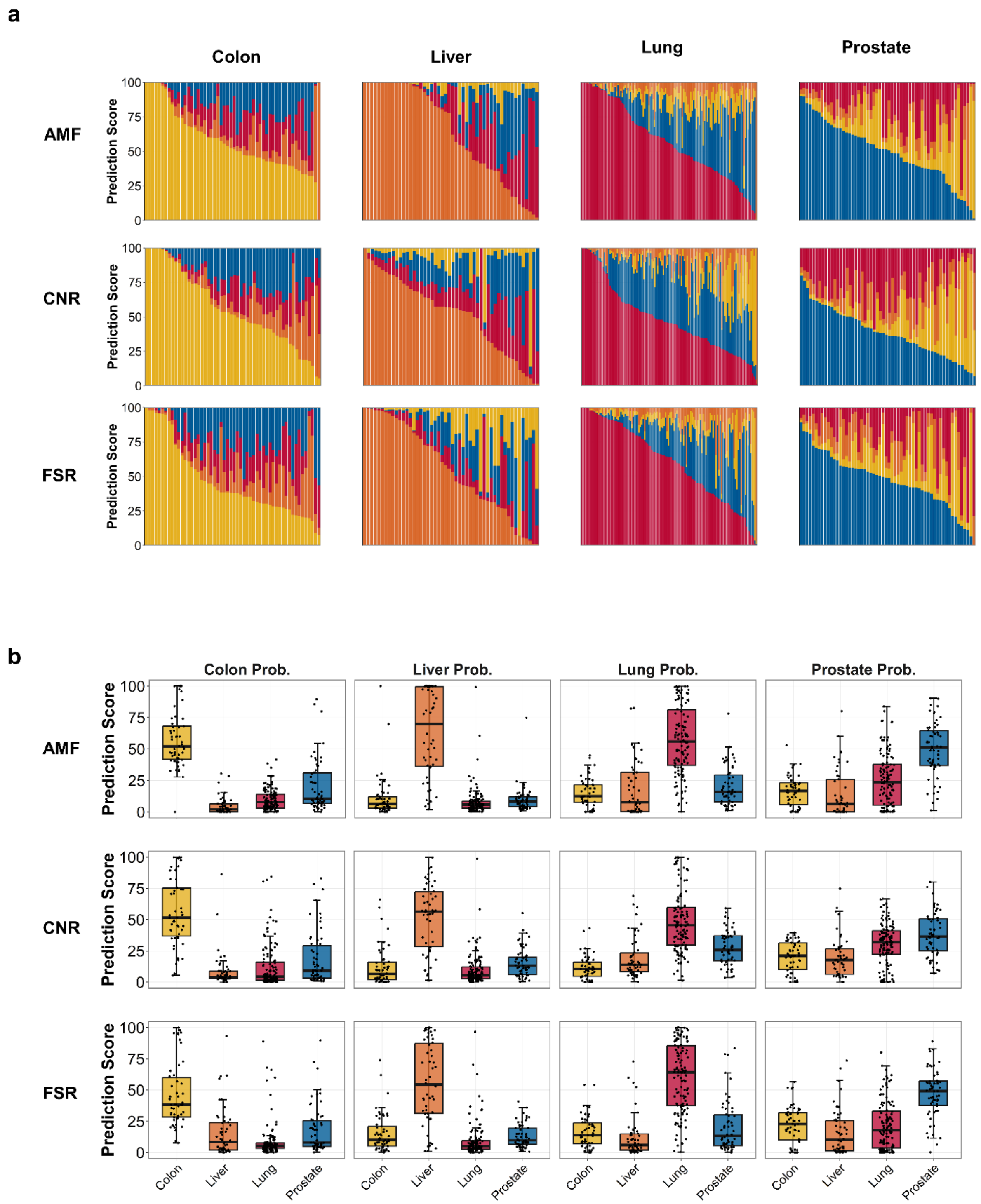

Supplementary Fig. 19. Distribution of the tissue of origin (TOO) conditional probability. **(a)** Stacked bar plot visualizing the conditional probability decomposition in each sample by average methylated fraction (AMF), copy number ratio (CNR), and fragment size ratio (FSR) TOO classifiers (rows) across the four cancer types (columns). **(b)** Boxplots showing each organ-supporting probability (titled in each panel) across the four cancer cohorts (x-axis).

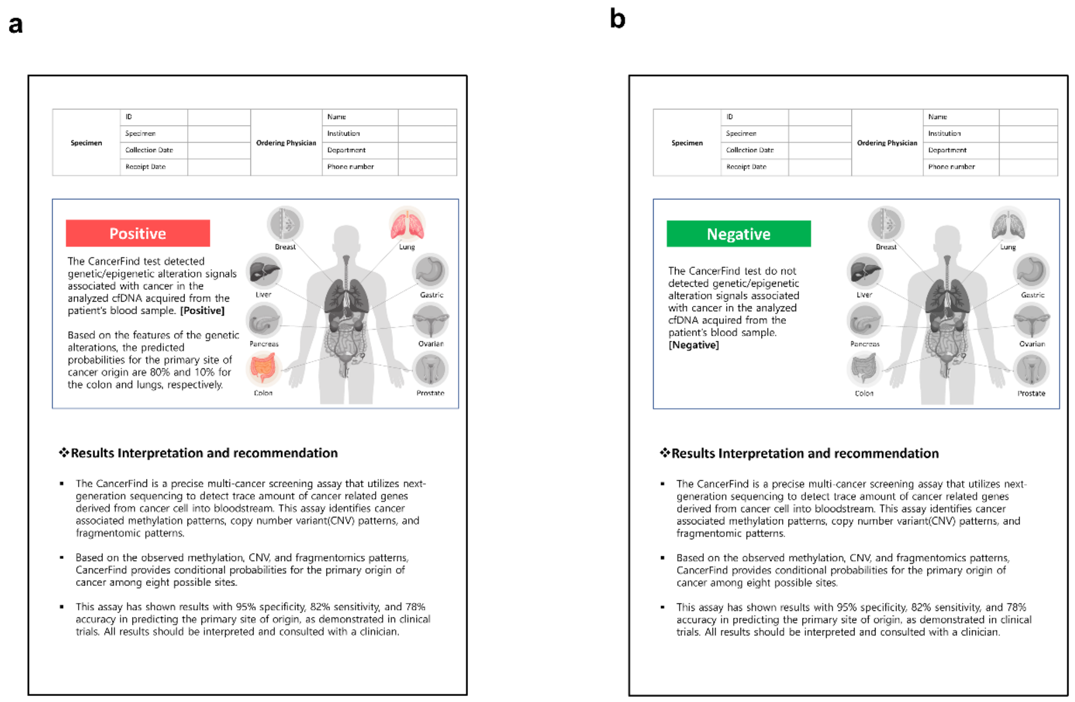

Supplementary Fig. 20. Draft version of AlphaLiquid® Screening platform report example. The report presents the positive/negative outcomes determined by the CSE model and the tissue-of-origin. Additionally, it encompasses a comprehensive interpretation of these findings accompanied by pertinent recommendations. (a) and (b) illustrate examples of a positive case and a negative case, respectively.
